# The enigma of eugregarine epicytic folds: where gliding motility originates?

**DOI:** 10.1186/1742-9994-10-57

**Published:** 2013-09-22

**Authors:** Andrea Valigurová, Naděžda Vaškovicová, Naďa Musilová, Joseph Schrével

**Affiliations:** 1 Department of Botany and Zoology, Faculty of Science, Masaryk University, Kotlářská 2, 611 37 Brno, Czech Republic; 2 Department of Biophysics, Faculty of Medicine, Masaryk University, Kamenice 3, 625 00 Brno, Czech Republic; 3 Muséum National d’Histoire Naturelle, UMR 7245 CNRS/MNHN, CP 52, 61 rue Buffon, 75231 Paris Cedex 05, France

**Keywords:** Actin, Cytochalasin D, Epicyte, Epicytic folds, Eugregarine, Glideosome, Gliding motility, Jasplakinolide, Mucus, Myosin, Pellicle

## Abstract

**Background:**

In the past decades, many studies focused on the cell motility of apicomplexan invasive stages as they represent a potential target for chemotherapeutic intervention. Gregarines (Conoidasida, Gregarinasina) are a heterogeneous group that parasitize invertebrates and urochordates, and are thought to be an early branching lineage of Apicomplexa. As characteristic of apicomplexan zoites, gregarines are covered by a complicated pellicle, consisting of the plasma membrane and the closely apposed inner membrane complex, which is associated with a number of cytoskeletal elements. The cell cortex of eugregarines, the epicyte, is more complicated than that of other apicomplexans, as it forms various superficial structures.

**Results:**

The epicyte of the eugregarines, *Gregarina cuneata*, *G. polymorpha* and *G. steini*, analysed in the present study is organised in longitudinal folds covering the entire cell. In mature trophozoites and gamonts, each epicytic fold exhibits similar ectoplasmic structures and is built up from the plasma membrane, inner membrane complex, 12-nm filaments, rippled dense structures and basal lamina. In addition, rib-like myonemes and an ectoplasmic network are frequently observed. Under experimental conditions, eugregarines showed varied speeds and paths of simple linear gliding. In all three species, actin and myosin were associated with the pellicle, and this actomyosin complex appeared to be restricted to the lateral parts of the epicytic folds. Treatment of living gamonts with jasplakinolide and cytochalasin D confirmed that actin actively participates in gregarine gliding. Contributions to gliding of specific subcellular components are discussed.

**Conclusions:**

Cell motility in gregarines and other apicomplexans share features in common, i.e. a three-layered pellicle, an actomyosin complex, and the polymerisation of actin during gliding. Although the general architecture and supramolecular organisation of the pellicle is not correlated with gliding rates of eugregarines, an increase in cytoplasmic mucus concentration is correlated. Furthermore, our data suggest that gregarines utilize several mechanisms of cell motility and that this is influenced by environmental conditions.

## Introduction

Apicomplexans are one of the most successful and diverse groups of eukaryotic unicellular parasites that exhibit unique adaptations to life in a wide spectrum of vertebrate and invertebrate hosts. Many cause major human diseases, i.e. malaria, toxoplasmosis, coccidiosis and cryptosporidiosis. Because apicomplexan diseases are still problematic, therapeutic research focuses either on parasitic structures or metabolic pathways which might serve as drug targets. The cytoskeleton of these parasites has become a focus for drug development because it plays an important role in various life processes, e.g., locomotion, division, invasion and formation of parasite cell polarity [1]. This is especially true of invasive stages of *Toxoplasma gondii* and *Plasmodium falciparum*[2,3].

Infective stages of Apicomplexa are characterised by an apical complex of organelles as well as a complicated cell cortex consisting of cortical alveoli, i.e., flattened vesicles limited by a membrane and packed into a continuous layer (inner membrane complex), underlying the plasma membrane. The inner membrane complex (IMC) has micropores and connects numerous cytoskeletal elements that include an actomyosin complex, microtubules and a network of intermediate filamentous proteins. The invasive zoites of Apicomplexa are motile and use actin-based gliding for host invasion and tissue traversal. This gliding mechanism called 'glideosome’ was first described for *Toxoplasma*[4] and has been extended as a concept to sporozoites of *Plasmodium*[5] and other apicomplexans [6]. In *Toxoplasma* and *Plasmodium*, myosin A is linked to the IMC and probably interacts with subpellicular microtubules. The head of myosin A moves along the actin filament, which is connected to a cell adhesion molecule (TRAP in *Plasmodium* spp. or TgMIC2 in *T. gondii*) via a tetrameric aldolase [6].

As deep-branching apicomplexan parasites of invertebrates and urochordates, gregarines (Gregarinasina) are generally thought to be of no economic importance. Recent analyses, however, indicate a close affinity of gregarines with species of *Cryptosporidium*[7,8] that parasitize humans. Most eugregarine gamonts are covered by a pellicle folded in numerous longitudinal epicytic folds (e.g., *Gregarina*, *Lecudina*) [9-11] and exhibit gliding motility [12-14]. Using a laser trap and bead translocation, King and Sleep [15] have described gliding as an irregular, erratic process. Some marine gregarines (e.g. archigregarines) possess regular sets of subpellicular microtubules under the pellicle [16,17] and typically display a pendular or rolling movement. In contrast, urosporidians that evolved as free-floating parasites within the host tissue move by pulsation of their body wall corresponding to the so-called peristaltic motility. The possible function of epicytic folds has been often discussed, and it is generally thought that they increase the surface area for nutrient acquisition and facilitate actomyosin-based gliding motility. The involvement of actin- and myosin-like proteins in gregarine cell motility has been previously reported [18-20]. Several electron microscopic studies have revealed the typical organisation of epicytic folds in eugregarines [10,11,21-23]. These suggest that there are undulating epicytic folds located between those that do not move [24-26], but the exact mechanism of motility remains unclear. Therefore, it is imperative that structural observations be integrated with biochemical and molecular data for the actin and myosins of *Gregarina* species [20,27]. Of the three myosin genes so far characterised in *Gregarina polymorpha*, myosins A (93 kDa) and B (96 kDa) belong to the class of myosin (XIV) that is restricted to the phylum Apicomplexa [28] and myosin F (222 kDa) to the class XII [27,29]. King and Sleep [15] estimated that the number of myosin heads at the site of interaction in *Gregarina* gamonts to be in excess of 200 and showed that the gliding rate in a giant eugregarine *Porospora gigantea* is four times higher (up to 60 μm/s) than the speed of myosin movement along the actin filaments of a muscle sarcomere. In spite of a few freeze-etching studies that focus on the supramolecular cell organisation of some *Gregarina* species [11,22,30], the precise location of the actomyosin complex is as yet unknown. However, the TRAP or TgMIC2 molecules that are in contact with the substrate suggest that the concept of a glideosome might help shed light on the role of the mucus [12] in the gliding mechanism of gregarines [31-33].

Laboratory-reared colony of the mealworm *Tenebrio molitor* parasitized by three species of *Gregarina* has permitted the comparison of gliding by *G. cuneata*, *G. polymorpha* and *G. steini* under identical environmental conditions. This study was performed so that the respective roles of the apical and lateral parts of the epicytic folds in apicomplexan zoite gliding could be discerned. Our intent was to evaluate the presumptive involvement of specific subcellular components such as the 12-nm filaments, rippled dense structures [11], and mucus in eugregarine gliding motility [32,33] using both the experimental and morphological approaches.

## Results

### Light microscopic observations on gregarine movement behaviour and gliding motility

**Figure 1:**
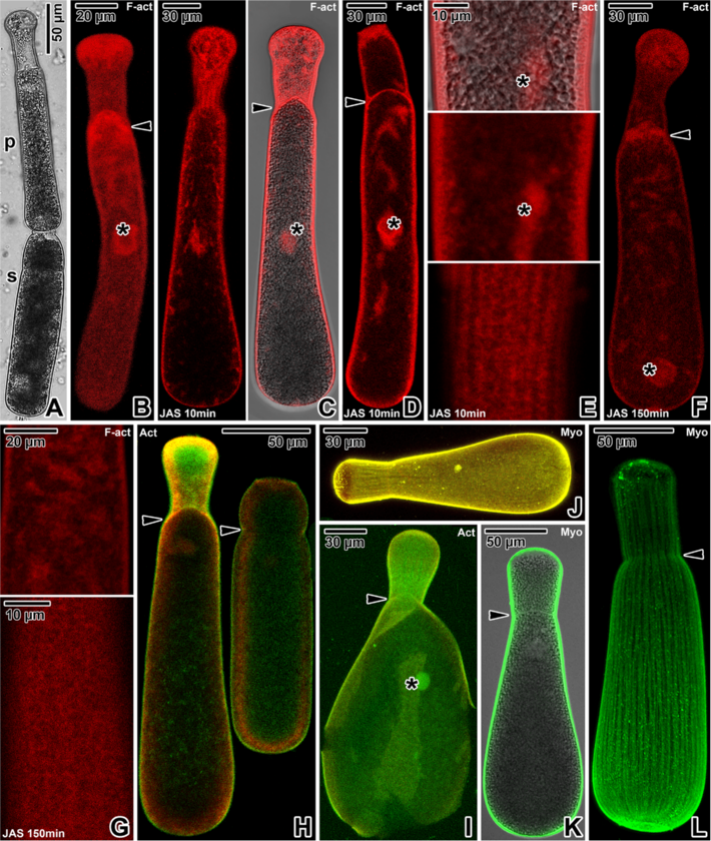
**Actin and myosin in *Gregarina cuneata *gamonts. A**. Gamonts in syzygy; primite (p), satellite (s). LM, transmitted light. **B**. Localisation of F-actin in a gamont; nucleus (asterisk), septum (arrowhead) between protomerite and deutomerite. CLSM, phalloidin-TRITC. **C-D**. Localisation of F-actin in gamonts (previously associated in syzygy) treated for 10 minutes with 10 μM JAS. Intense labelling is restricted to the cortex and cytoplasmic F-actin aggregations; septum (arrowhead), nucleus (asterisk). Figure **C** shows a primite. CLSM (left) and merged CLSM/transmitted light (right), phalloidin-TRITC. Figure **D** shows a satellite. CLSM, phalloidin-TRITC. **E**. The deutomerite of a gamont treated for 10 minutes with 10 μM JAS. F-actin localisation corresponds to the cortex and nucleus (asterisk). Upper two figures show the gamont middle plane; lower figure shows the cortex in the area of epicytic folds. Merged CLSM/transmitted light (upper) and CLSM, phalloidin-TRITC. **F**. F-actin localisation in a gamont treated for 150 minutes with 10 μM JAS. Note the decreased labelling of cell cortex and septum (arrowhead), and formation of numerous cytoplasmic aggregations of F-actin; nucleus (asterisk). CLSM, phalloidin-TRITC. **G**. The deutomerite of a gamont treated for 150 minutes with 10 μM JAS. F-actin labelling is restricted to the cortex in the area of epicytic folds (lower); cytoplasmic F-actin aggregations (upper). CLSM, phalloidin-TRITC. **H**. Actin localisation in previously associated gamonts; septum (arrowheads). CLSM, IFA. **I**. Actin localisation in a gamont ghost; nucleus (asterisk), septum (arrowhead). CLSM, IFA. **J**. Myosin labelling in a maturing gamont. CLSM, IFA. **K**. Myosin labelling in a mature gamont is restricted to the cortex, but not to the septum (arrowhead). Merged CLSM/transmitted light, IFA. **L**. Labelling of myosin in a gamont cortex shows a pattern of longitudinal rows; septum (arrowhead). CLSM, IFA. Figures **H**, **I** and **J** show merged FITC (antibody) and rhodamine (counterstaining with Evans blue) fluorescence channels.

**Figure 2:**
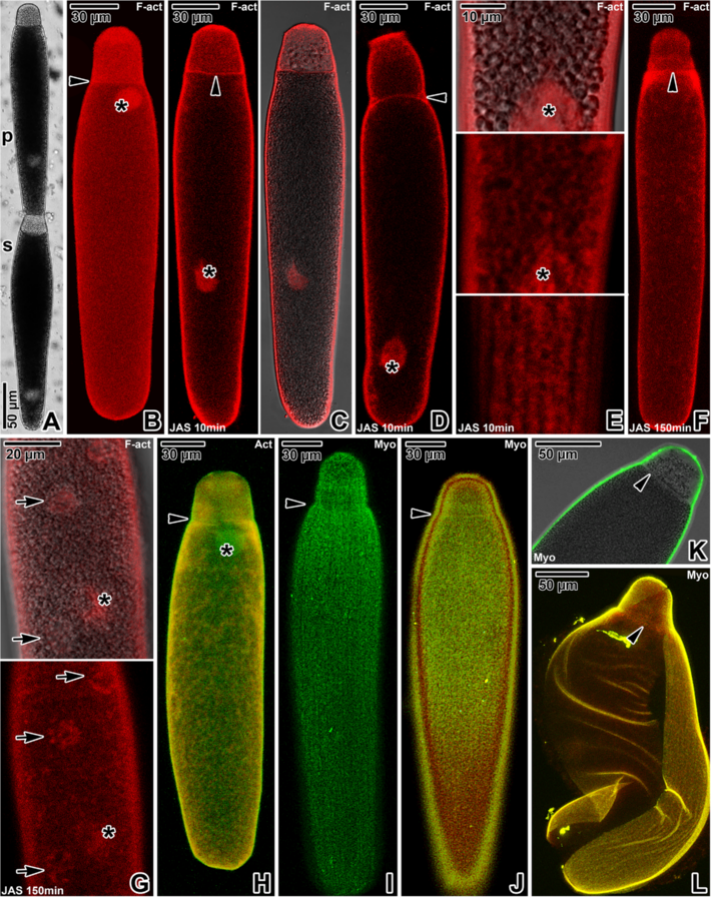
**Actin and myosin in *Gregarina polymorpha *gamonts. A**. Gamonts in syzygy; primite (p), satellite (s). LM, transmitted light. **B**. F-actin localisation in a gamont; nucleus (asterisk), septum (arrowhead). CLSM, phalloidin-TRITC. **C-D**. F-actin localisation in gamonts (previously associated in syzygy) treated for 10 minutes with 10 μM JAS. The more intense labelling is restricted to the cortex; septum (arrowhead), nucleus (asterisk). Figure **C** shows a primite. CLSM (left) and merged CLSM/transmitted light (right), phalloidin-TRITC. Figure **D** shows a satellite. CLSM, phalloidin-TRITC. **E**. The deutomerite of a gamont treated for 10 minutes with 10 μM JAS. F-actin localisation corresponds to the cortex; nucleus (asterisk). Upper two figures show the gamont middle plane; lower figure shows the cortex in the area of epicytic folds. Merged CLSM/transmitted light (upper) and CLSM, phalloidin-TRITC. **F**. F-actin localisation in a gamont treated for 150 minutes with 10 μM JAS, showing decreased labelling of cortex and septum (arrowhead). Numerous cytoplasmic F-actin aggregations give the labelling homogenous appearance. CLSM, phalloidin-TRITC. **G**. The deutomerite of a gamont treated for 150 minutes with 10 μM JAS; rosette-like aggregations of F-actin (arrows), nucleus (asterisk). Merged CLSM/transmitted light (upper) and CLSM (lower), phalloidin-TRITC. **H**. Actin localisation in a gamont; nucleus (asterisk), septum (arrowhead). The indistinct labelling (green) is more evident in the cortex covering the anterior part of cell. CLSM, IFA. **I–J**. Myosin localisation in a gamont. The labelling is restricted to the cortex, with a pattern of longitudinal rows; septum (arrowhead). CLSM, IFA. **K**. The anterior part of the gamont shown in J. Myosin localisation is restricted to the cortex, but not to the septum (arrowhead). Merged CLSM/transmitted light, IFA. **L**. Myosin localisation in a gamont ghost; septum (arrowhead). CLSM, IFA. Figures **H**, **J** and **L** show merged FITC (antibody) and rhodamine (counterstaining with Evans blue) fluorescence channels.

**Figure 3:**
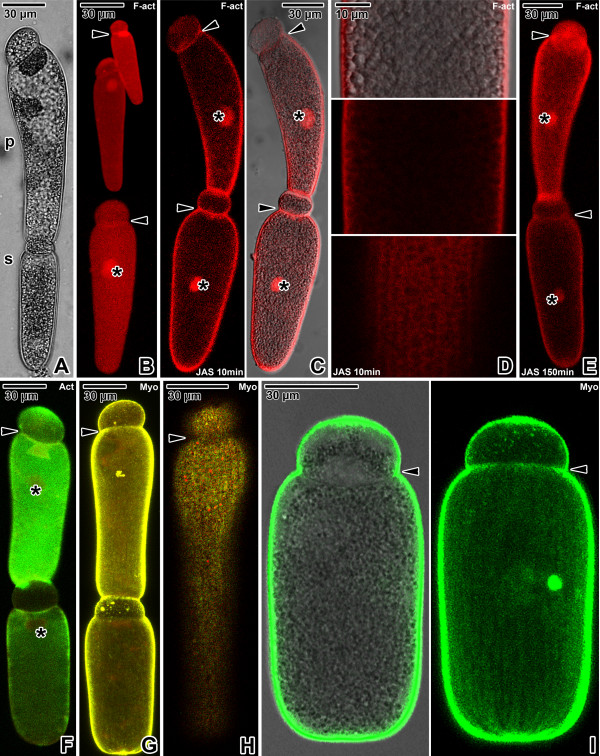
**Actin and myosin in *Gregarina steini *gamonts. A**. Gamonts associated in syzygy; primite (p), satellite (s). LM, transmitted light. **B**. Localisation of F-actin in three single gamonts; nucleus (asterisk), septum (arrowheads). CLSM, phalloidin-TRITC. **C**. Localisation of F-actin in gamonts associated in syzygy treated for 10 minutes with 10 μM JAS. The intense labelling is restricted to the cortex, septum (arrowheads) and nucleus (asterisks). CLSM (left) and merged CLSM/transmitted light (right), phalloidin-TRITC. **D**. The deutomerite of a gamont treated for 10 minutes with 10 μM JAS. The localisation of F-actin corresponds to the cortex. Upper two figures show the gamont middle plane; lower figure is a view of the cortex in the area of epicytic folds. Merged CLSM/transmitted light (upper) and CLSM, phalloidin-TRITC. **E**. Localisation of F-actin in gamonts associated in syzygy treated for 150 minutes with 10 μM JAS. Note the decreased labelling of cell cortex and septum (arrowheads); nucleus (asterisks). Numerous indistinct, small cytoplasmic aggregations of F-actin give the labelling a more homogenous appearance. CLSM, phalloidin-TRITC. **F**. Actin localisation in gamonts associated in syzygy; nucleus (asterisks). The septum (arrowhead) exhibits an intense labelling. CLSM, IFA. **G**. Myosin labelling in gamonts associated in syzygy; septum (arrowhead). CLSM, IFA. **H**. An optical plane of the gamont cortex showing the distribution of myosin corresponding to the epicytic folds; septum area (arrowhead). CLSM, IFA. **I.** Myosin localisation in a single gamont. The labelling is restricted to the cortex and exhibits a pattern of longitudinal rows. A slight labelling in the septum area is shown (arrowhead). Merged CLSM/transmitted light (left) and CLSM (right), IFA. Figures **F**, **G** and **H** show merged FITC (antibody) and rhodamine (counterstaining with Evans blue) fluorescence channels.

The pellicle (epicyte) appeared as a thick but transparent layer of even width covering the entire gregarine (Figures 1A, 2A and 3A). Longitudinal striations that were easily recognisable in the pellicle corresponded to the epicytic folds. During gliding, the shape of the cell varied by species. In *G. cuneata* and *G. polymorpha*, the changes of direction during gliding seemed to be controlled by protomerite activity. In *G. polymorpha* the protomerite and deutomerite were very flexible. The bending of the protomerite may take place in any plane, but in *G. polymorpha* it was sometimes so extensive that the axis of the protomerite (or even with the one-third of the deutomerite) came to form a right or even acute angle with that of the deutomerite. This behaviour was especially noted when gamonts encountered barriers in their gliding path. In *G. polymorpha*, a partial pulling of the protomerite into the deutomerite, so that the corresponding pellicle became pleated, could be observed. A slight bending of the protomerite could be detected also in *G. cuneata*, but the angle between the planes of protomerite and deutomerite was only obtuse. In contrast, the protomerite of *G. steini* did not show any changes during gliding; only a slight bending of the gamont deutomerite, usually in its posterior half, was observable when turning to the side. In syzygies of all species, the satellite seemed to be passive and just followed the path given by the obviously active primite, and this path corresponded to a forward unidirectional gliding. In all three species, the gliding locomotion of single and associated gamonts was usually discontinuous with multiple stops and frequent changes of direction, and often occurred with discernible changes in speed. The gregarines glided in an almost linear pattern. The gliding movement, however, was not constant and varied among species as well as every gliding individual. The maximum speed of gliding gamonts achieved during our observations was 5.66 μm/s in single gamonts and 8.49 μm/s in syzygies of *G. cuneata,* 22.86 μm/s in single gamonts and 16.18 μm/s in syzygies of *G. polymorpha*, and 9.42 μm/s in single gamonts and 9.25 μm/s in syzygies of *G. steini* (Table 1). Based on contact stimuli, gregarines were able to quite rapidly change the direction of their otherwise straightforward gliding to avoid a barrier in their gliding path. When a gliding gamont encountered a barrier, it usually endeavoured to bore or wriggle its way through. Obviously, gamonts of *G. steini* exhibited the most continuous and constant gliding with a linear or widely semi-circular track. The gliding of *G. cuneata* gamonts was characterised by multiple and prolonged stops, and thus many individuals did not exhibit gliding during the recording. In contrast, gamonts of *G. polymorpha* covered the greatest distance per unit of time in one continuous track.

**Table 1:** The speed in observed gliding gregarines

**Species**	**Gliding speed in single gamonts (μm/s)**					**Gliding speed in syzygies (μm/s)**				
	**Minimum - maximum**	**Mean**	**Standard deviation**	**N. of gamonts**	**N. of records**	**Minimum - maximum**	**Mean**	**Standard deviation**	**N. of syzygies**	**N. of records**
***G. cuneata***	0.38 - 5.66	2.20	1.51	18	24	1.15 - 8.49	3.96	2.47	8	8
***G. polymorpha***	3.46 - 22.86	9.91	5.15	12	16	1.05 - 16.18	6.73	4.08	14	17
***G. steini***	1.32 - 9.42	5.02	2.19	33	39	2.51 - 9.25	5.33	1.62	23	23

### Treatment of gregarine gamonts with jasplakinolide and cytochalasin D

Various concentrations of both drugs were used to treat gregarine gamonts. Concentrations of jasplakinolide (JAS), a strong actin stabilizer, lower than 5 μM had no effect. Thus, in order to obtain reliable results on active and vital gregarines, they were treated with 5, 10, 20 and 30 μM concentrations of JAS in Ringer’s solution. Gregarines not only survived in these very high doses of JAS, but even actively moved for the next 90–150 min, depending on the drug concentration and gregarine species (Table 2). Experiments revealed that JAS treatment led to an increased speed of gliding movement beyond 5 minutes after drug application, followed by subsequent decrease to normal in all three species. Afterwards, individual reactions rates to JAS differed by species with the most rapid occurring in *G. steini* whose gamonts moved up to 90 minutes. The most delayed reaction to the drug (inhibition of gliding motility) exhibited syzygies of *G. cuneata*, which moved in large numbers up to 150 minutes after JAS application. After a uniform period of 1 hour, independently on above mentioned concentrations of JAS, all three species exhibited obvious cellular changes including shrivelling and some degree of cytoplasmic disorganisation. In all species, cell shape restoration took place immediately after returning gamonts to the Ringer’s solution. Nevertheless, the time needed for full recovery of gregarines along with the restoration of their motility varied by species (or even individuals) and applied JAS concentrations. The most rapid recovery has been observed in *G. cuneata*. In contrast, gamonts of *G. steini* needed much longer time, and on top of that, some of them did not survive the experiment. In all control preparations, the gamonts continued to move until the end of the experiment. Interestingly, during the first 30 minutes, in contrast to *G. polymorpha* and *G. steini*, gamonts of *G. cuneata* moved more rapidly in a drop of Ringer’s solution than was observed on microbiological agar only slightly moistened with Ringer’s solution. Although their movement in this period resembled regular gliding in contact with the substrate, detailed observations revealed that they were rather free-floating in a liquid. After 30 minutes, gamonts of all three species sank to the surface of the microscopic slide and started to glide in a regular way, exhibiting the same speed of movement as observed during the motility experiments performed on moistened agar or in the Bürker counting chamber described above (Table 1).

**Table 2:** The treatment of living gregarines with jasplakinolide

**Species/JAS concentration**	***Gregarina cuneata***				***Gregarina polymorpha***				***Gregarina steini***			
	**5 μM JAS**	**10 μM JAS**	**20 μM JAS**	**30 μM JAS**	**5 μM JAS**	**10 μM JAS**	**20 μM JAS**	**30 μM JAS**	**5 μM JAS**	**10 μM JAS**	**20 μM JAS**	**30 μM JAS**
**Changes/time left after drug application (in minutes)**												
**Initial increase of gliding speed**	≥ 5 min+	≥ 5 min+	≥ 5 min++	≥ 5 min++	≥ 5 min+	≥ 5 min+	≥ 5 min++	≥ 5 min++	≥ 5 min+	≥ 5 min+	≥ 5 min++	≥ 5 min++
**Decrease of gliding speed to the normal rate**	≥ 90 min+	≥ 20 min++	≥ 20 min++	≥ 20 min++	≥ 90 min+	≥ 20 min++	≥ 20 min++	≥ 20 min++	≥ 90 min+	≥ 20 min++	≥ 20 min++	≥ 20 min++
**Further decrease of gliding speed**	≥ 100 min+	≥ 30 min++	≥ 30 min++	≥ 30 min++	≥ 100 min+	≥ 30 min++	≥ 30 min++	≥ 30 min++	≥ 100 min+	≥ 30 min+	≥ 30 min++	≥ 30 min++
**Cellular changes (shrivelling)**	≥ 60 min	≥ 60 min	≥ 60 min	≥ 60 min	≥ 60 min	≥ 60 min	≥ 60 min	≥ 60 min	≥ 60 min	≥ 60 min	≥ 60 min	≥ 60 min
**Complete stoppage of gliding motility**	≤ 150 min	≤ 140 min	≤ 100 min	≤ 100 min	≤ 150 min	≤ 140 min	≤ 100 min	≤ 100 min	≤ 130 min	≤ 120 min	≤ 90 min	≤ 90 min
**Recovery of cell shape after washing in Ringer’s solution**	≤ 1 min	≤ 1 min	≤ 1 min	≤ 1 min	≤ 1 min	≤ 1 min	≤ 1 min	≤ 1 min	≤ 1 min	≤ 1 min	≤ 1 min	≤ 1 min
**Full recovery of motility after washing in Ringer’s solution**	≥ 5 min	≥ 5 min	≥ 5 min	≥ 5 min	≤ 10 min	≥ 10 min	≥ 10 min	≥ 10 min	≤ 30 min	≥ 30 min	≥ 30 min	≥ 30 min

The symbol + indicates an intensity of observed change ranging from + to +++ when compared among gregarine species and JAS concentrations. Applied only if the listed phenomenon showed significant differences in its intensity.

Treatments with cytochalasin D (10, 20 and 30 μM in Ringer’s solution), an inhibitor of actin polymerisation, completely inhibited gregarine motility in a species- and concentration dependent manner; i.e. at 10-30 minutes in *G. steini*, 30-75 minutes in *G. polymorpha* and 75-120 minutes in *G. cuneata*. The cellular changes, observed in all assays after a uniform period of 30 minutes, were less obvious than those induced by JAS. In all species, cell shape restoration took place immediately after returning to the Ringer’s solution. The time needed for full recovery and restoration of gregarine motility varied by species and drug concentrations (Table 3).

**Table 3:** The treatment of living gregarines with cytochalasin D

**Species/Cytochalasin D concentration**	***Gregarina cuneata***			***Gregarina polymorpha***			***Gregarina steini***		
	**10 μM CytD**	**20 μM CytD**	**30 μM CytD**	**10 μM CytD**	**20 μM CytD**	**30 μM CytD**	**10 μM CytD**	**20 μM CytD**	**30 μM CytD**
**Changes/time left after drug application (in minutes)**									
**Initial decrease of gliding speed**	≥ 5 min+	≥ 5 min++	≥ 5 min+++	≥ 5 min+	≥ 5 min++	≥ 5 min+++	≥ 5 min+	≥ 5 min++	≥ 5 min+++
**Further decrease of gliding speed**	≥ 20 min+	≥ 10 min++	≥ 10 min+++	≥ 15 min+	≥ 10 min++	≥ 10 min+++	≥ 10 min+	≥ 10 min++	≥ 10 min+++
**Cellular changes (shrivelling)**	≥ 30 min	≥ 30 min	≥ 30 min	≥ 30 min	≥ 30 min	≥ 30 min	≥ 30 min	≥ 30 min	≥ 30 min
**Complete stoppage of gliding motility**	≤ 120 min	≤ 90 min	≤ 75 min	≤ 75 min	≤ 60 min	≤ 30 min	≤ 30 min	≤ 20 min	≤ 10 min
**Recovery after washing in Ringer’s solution**	≤ 1 min	≤ 1 min	≤ 1 min	≤ 1 min	≤ 1 min	≤ 1 min	≤ 1 min	≤ 1 min	≤ 1 min
**Full recovery after washing in Ringer’s solution**	≥ 5 min	≤ 10 min	≤ 10 min	≥ 5 min	≤ 10 min	≤ 10 min	≥ 5 min	≥ 10 min	≥ 10 min

The symbol + indicates an intensity of observed change ranging from + to +++ when compared between gregarine species and concentrations of cytochalasin D. Applied only if the listed phenomenon showed significant differences in its intensity.

### Confocal microscopic analysis of actomyosin motor

In all three species, the homogenous distribution of the fluorescence signal throughout the surface of phalloidin- and antibody-labelled gamonts corresponded to the localisation of an actomyosin motor associated with the apicomplexan cell cortex. Phalloidin labelling confirmed the presence of filamentous actin (F-actin) in the gregarine cell cortex, the fibrillar septum separating the protomerite from the deutomerite and the area of the nucleus (Figures 1B, 2B and 3B). After treatment with 10 μM JAS for 10 minutes, when gregarines glided with increased speed, the F-actin staining became more bright and confined to the cell cortex, the septum and the perinuclear space (Figures 1C-E, 2C-E and 3C-D). Higher magnification revealed numerous transverse and oblique actin filaments in the area of epicytic folds (Figures 1E, 2E and 3D). In addition, several aggregations of F-actin were observed in the cytoplasm of *G. cuneata* protomerite and deutomerite (Figure 1C-D). This was accompanied by an obvious decrease of diffuse F-actin in all analysed gregarines. The localisation of F-actin did not significantly change even after 150 minutes incubation in 10 μM JAS, when gregarines completely stopped their movement and showed obvious cellular changes, but the intensity of cell cortex labelling significantly decreased and numerous small aggregations of F-actin appeared in the cell cytoplasm (Figures 1F-G, 2F-G and 3E). The transverse actin filaments in epicytic folds appeared to fuse into a homogeneous layer. In *G. polymorpha*, rosette-like aggregations of F-actin were situated in the cell periphery (Figure 2G).

Gamonts stained with the specific anti-actin antibody (known to recognise the actin in *Toxoplasma* and *Plasmodium*) exhibited a similar actin localisation, however, in comparison with the phalloidin-stained specimens, only slight labelling of actin was associated with the cell cortex and the septum in *G. cuneata* (Figure 1H–I) and *G. polymorpha* (Figure 2H). Gamonts of *G. steini* exhibited a higher intensity of actin labelling with this antibody, and gamonts associated in syzygy labelled with a different intensity in that a higher concentration of actin was observed in the primite (Figure 3F).

Similarly to actin, the myosin was restricted to the cell cortex (Figures 1J–L, 2I–L and 3G–I), but no specific labelling corresponding to the septum was observed. When focussing on the gregarine surface, the organisation of myosin exhibited a pattern of longitudinal rows corresponding to the epicytic folds (Figures 1L, 2I–J and 3H-I). The primites and satellites in syzygies (Figure 3G) exhibited more or less identical intensity of labelling.

The results of immunofluorescent labelling using an anti-α-tubulin antibody were negative (data not shown) in agreement with the absence of subpellicular microtubules.

### Mucus shedding

**Figure 4:**
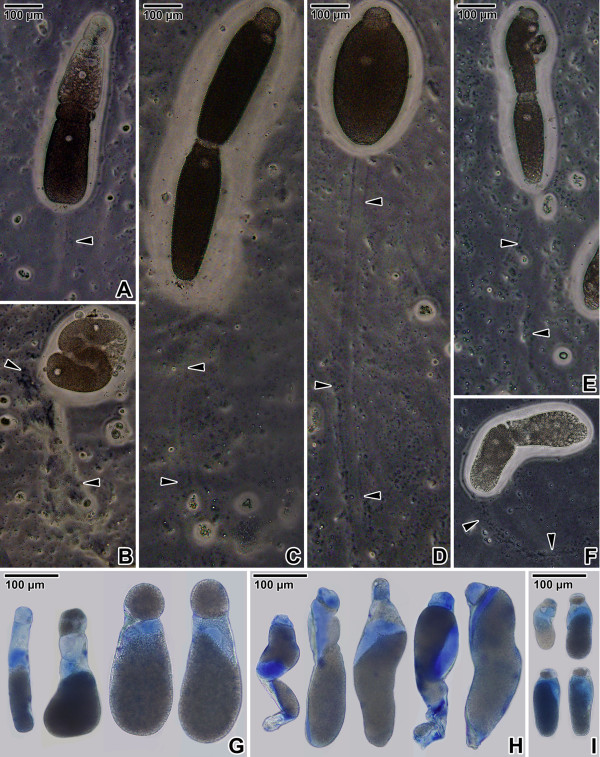
**Gliding motility and mucus. A–F**. Mucous trail (arrowheads) left behind gliding gregarines; syzygy of *Gregarina cuneata***(A, B)**, syzygy **(C)** and single gamont **(D)** of *Gregarina polymorpha*, syzygy of *Gregarina steini***(E, F)**. Note the increased mucus shedding by the syzygy of *G. cuneata* exhibiting rotary movement **(B)**. **G–I**. Light micrographs showing the volume of mucus in gamonts of *G. cuneata***(G)**, *G. polymorpha***(H)** and *G. steini***(I)** revealed with Alcian blue staining at low pH.

Only phase contrast microscopy was able to show mucus shedding and trail formation of gamonts gliding on agar (Figure 4A–F). The most evident mucous trails were that of associated (Figure 4C) or single (Figure 4D) gamonts of *G. polymorpha* due to the distance travelled by them, which left long and regular mucous paths. In contrast, *G. cuneata* (Figure 4A) and *G. steini* (Figure 4E) gliding gamonts exhibited irregular and short paths containing greater accumulations of mucus, especially when these species were avoiding barriers or altering direction (Figure 4B and F).

The mucous substances were visualized by Alcian blue staining within the gamont cytoplasm (Figure 4G–I). The most intense labelling occurred in the deutomerite cytoplasm of *G. polymorpha* (Figure 4H). In contrast to *G. cuneata* (Figure 4G) and *G. steini* (Figure 4I), this gregarine showed an increased volume of mucus in the protomerite cytoplasm, which might be correlated with the increased motility of the *G. polymorpha* protomerite.

### Ultrastructural features of the pellicle

**Figure 5:**
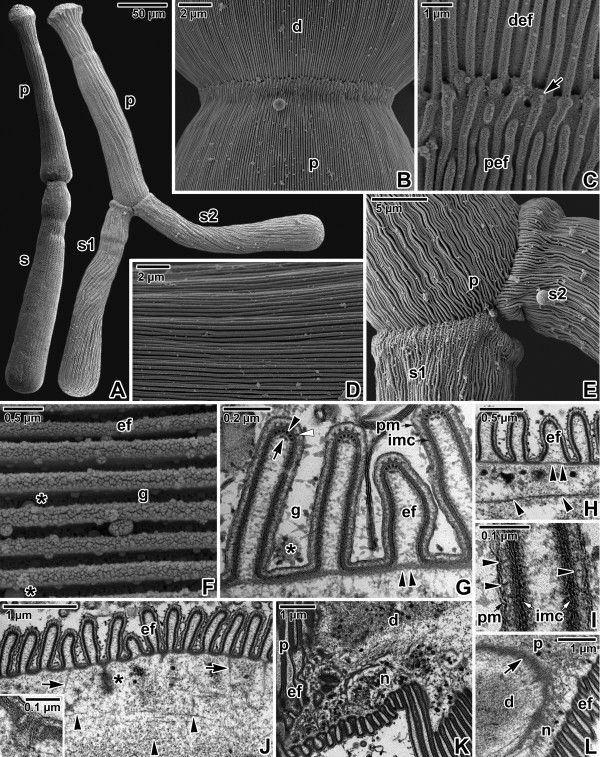
**Pellicle architecture in *Gregarina cuneata *gamonts. A**. Gamonts associated in syzygy; primite (p), satellite (s). SEM. The syzygy on the right is composed of one primite (p) and two satellites (s1, s2). **B**. Higher magnification of the junction between the posterior end of the primite deutomerite (d) and the apical part of the satellite protomerite (p). SEM. **C**. A detail of the junction (arrow) between folded pellicles covering the primite deutomerite (def) and satellite protomerite (pef). SEM. **D**. Organisation of linear epicytic folds covering the deutomerite. SEM. **E**. Higher magnification of the junction between the primite (p) and two satellites (s1, s2) shown in panel A. SEM. **F**. A higher magnification of deutomerite epicytic folds (ef); grooves (g) between folds, mucus drops (*). SEM. **G**. Cross section of deutomerite epicytic folds; grooves (g) with mucus (*) between folds (ef), 12-nm filaments (arrowhead), inner membrane complex (imc), internal lamina (double arrowhead), plasma membrane (pm), rippled dense structures (white arrowhead), unknown dense structure (arrow). TEM. **H**. Cross section of deutomerite epicytic folds (ef); internal lamina (double arrowhead), rib-like myonemes (arrowheads). TEM. **I**. Detailed view of an epicytic fold in cross section revealing filamentous connections (arrowheads) localised between the plasma membrane (pm) and inner membrane complex (imc). TEM. **J**. Cross section showing the organisation of the deutomerite pellicle; ectoplasmic network (arrows), epicytic folds (ef), duct (*), rib-like myonemes (arrowheads). The insect shows the micropore located in the groove between two epicytic folds. TEM. **K**. Organisation of the pellicle and ectoplasmic network (n) during gregarine movement; epicytic folds (ef), deutomerite (d), protomerite (p). TEM. **L**. Higher magnification of a septum (arrow) separating the protomerite (p) from the deutomerite (d); epicytic folds (ef). Note the ectoplasmic network (n) connected to the septum. TEM.

**Figure 6:**
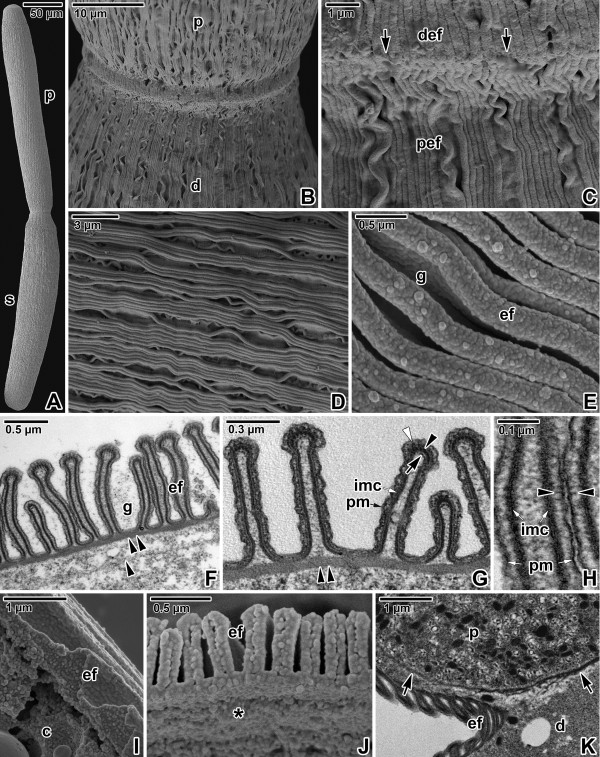
**Pellicle architecture in *Gregarina polymorpha *gamonts. A**. Gamonts associated in syzygy; primite (p), satellite (s). SEM. **B**. Higher magnification of the junction between the posterior end of the primite deutomerite (d) and apical part of the satellite protomerite (p). SEM. **C**. Detailed view of the junction (arrows) between folded pellicles covering the primite deutomerite (def) and the satellite protomerite (pef). SEM. **D**. Organisation of undulated epicytic folds covering the deutomerite. SEM. **E**. Higher magnification of deutomerite epicytic folds (ef); grooves (g) between folds. SEM. **F**. Cross section of the deutomerite pellicle; epicytic folds (ef), grooves (g), internal lamina (double arrowhead), rib-like myonemes (arrowhead). TEM. **G**. Cross section of deutomerite epicytic folds; 12-nm filaments (arrowhead), inner membrane complex (imc), internal lamina (double arrowhead), plasma membrane (pm), rippled dense structures (white arrowhead), unknown dense structure (arrow). TEM. **H**. Detailed view of epicytic folds in cross section revealing filamentous connections (arrowheads) localised between the plasma membrane (pm) and inner membrane complex (imc). TEM. **I**. Pellicle organisation revealed in a mechanically ruptured gamont; cytoplasm (c) with ectoplasmic network, epicytic folds (ef). SEM. **J**. The view of an ectoplasmic face (*) of a pellicle separated from the gamont cytoplasm; epicytic folds (ef). SEM. **K**. The septum (arrow) separating the protomerite (p) from the deutomerite (d); epicytic folds (ef). TEM.

**Figure 7:**
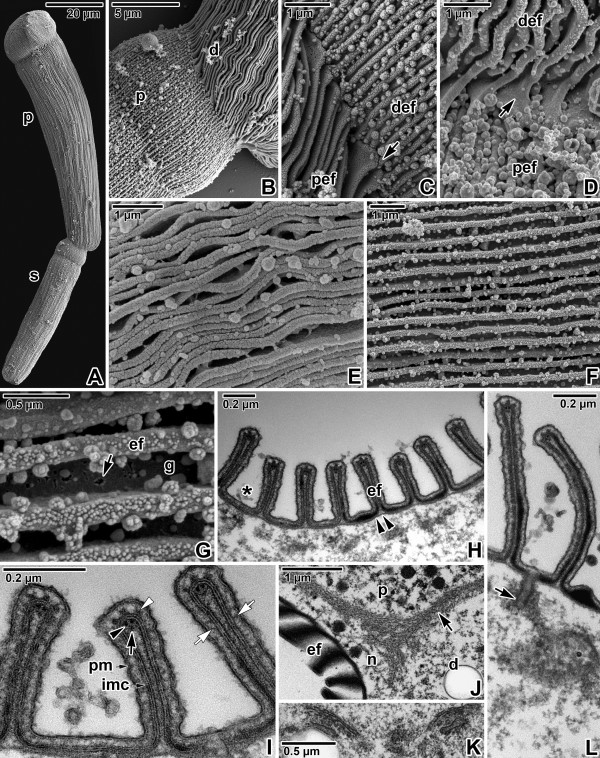
**Pellicle architecture in *Gregarina steini *gamonts. A**. Gamonts associated in syzygy; primite (p), satellite (s). SEM. **B**. Higher magnification of the junction between the posterior end of the primite deutomerite (d) and apical part of the satellite protomerite (p). SEM. **C**, **D**. Detailed views of the junction (arrow) between folded pellicles covering the primite deutomerite (def) and satellite protomerite (pef). SEM. **E**, **F**. Organisation of more and less undulated epicytic folds covering the deutomerite. SEM. **G**. Higher magnification of deutomerite epicytic folds (ef); grooves (g) between folds, pore-like structure (arrow). SEM. **H**. Organisation of the deutomerite pellicle in cross section; epicytic folds (ef), grooves with mucus (*), internal lamina (double arrowhead). TEM. **I**. Cross section of deutomerite epicytic folds. Note the filamentous connections (white arrows) localised between the plasma membrane (pm) and inner membrane complex (imc); 12-nm filaments (arrowhead), rippled dense structures (white arrowhead), unknown dense structure (arrow). TEM. **J**. The septum (arrow) and ectoplasmic network (n); deutomerite (d), epicytic folds (ef), protomerite (p). TEM. **K**. Golgi apparatus in deutomerite cytoplasm. TEM. **L**. The duct (arrow) opening outwards to the groove between epicytic folds. TEM.

The pellicle in all the species was organised in numerous longitudinal narrow folds, which were more raised in the deutomerite region than in the protomerite. The epicytic folds of the deutomerite were more or less undulated, depending on the species. Similarly, the number of epicytic folds per square micrometer varied among species, and their number did not significantly changed in the course of gamont growth (Table 4). The gamonts *G. cuneata* were covered by almost linear folds and numerous mucus-like drops were often present in the grooves separating them (Figure 5A–F). Occasionally, rings of undulated folds could be observed, especially in the posterior half of the gamont deutomerite (Figure 5E). The pellicle appeared to be slightly helically coiled along the gregarine longitudinal axis, resulting in a helical course of epicytic folds (Figure 5A, the syzygy on the right). The pellicle of *G. polymorpha* gamonts exhibited zones of almost linear folds alternating with zones of much undulated folds; however, surprisingly when considering the results of the Alcian blue staining, only a few mucus drops could be detected (Figure 6A–E). The epicytic folds of *G. steini* were undulated in a more regular pattern than in *G. polymorpha,* and numerous mucus-like drops covered the entire surface of the gamonts (Figure 7A–G). Depending on the species, more or less evident constriction could be found at the interface between the protomerite and deutomerite; however, no interruption of folds, running from the gamont apical to its posterior end, was present in this area (Figures 5A, 6A and 7A). Syzygies were caudo-frontal, i.e. the posterior end of the primite deutomerite was joined with the apical part of the satellite protomerite (Figures 5A, 6A and 7A). The connection area appeared as a collar-like junction composed of modified epicytic folds of the primite deutomerite meshing in a gear-like manner with the folds of the sucker-like apical region of the satellite (Figures 5B–C, 6B–C and 7B–D). In *G. cuneata*, two or more satellites were often found to be associated with one primite (Figure 5A and E). In some cases, a large primite was associated with several tiny satellites (up to four satellites associated with one primite were observed). Occasionally, three individuals of *G. cuneata* were seen to be associated in a row, the last one of which was the smallest.

**Table 4:** The number of epicytic folds in deutomerite of gamonts

**Species**	***G. cuneata***		***G. polymorpha***		***G. steini***	
**Gamont N.**	**Number of folds/μm**^**2**^	**Deutomerite perimeter** (**μm)**	**Number of folds/μm**^**2**^	**Deutomerite perimeter (μm)**	**Number of folds/μm**^**2**^	**Deutomerite perimeter (μm)**
**1**	4.5	53.4	4.3	94.3	5.6	31.4
**2**	4.4	98.1	4.2	103.7	6.3	34.6
**3**	3.9	113.1	4.9	106.8	6.3	37.7
**4**	3.6	114.0	4.3	110.0	5.9	58.0
**5**	3.2	150.9	4.5	132.0	5.0	66.0
**6**	3.3	163.4	3.6	144.5	4.0	113.1

**Figure 8:**
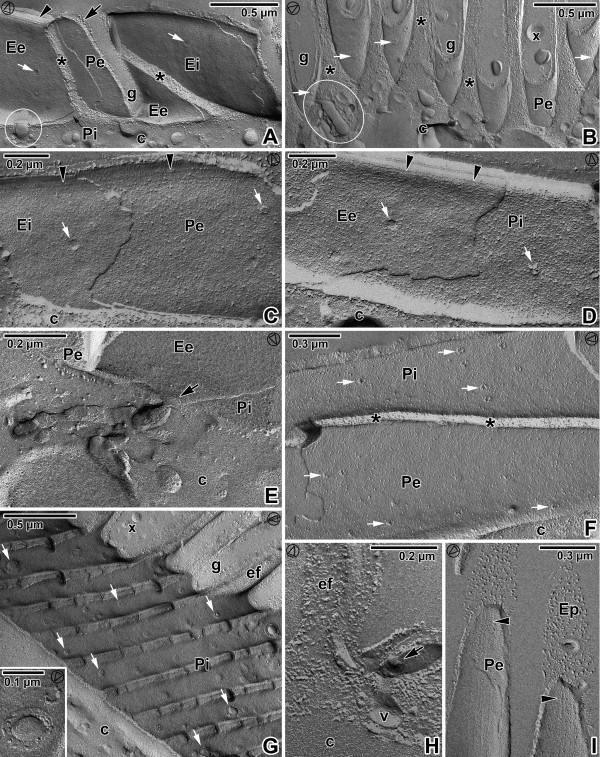
**Pellicle organisation in *Gregarina cuneata *gamonts as revealed by the freeze-etching. A**. The general view of fractured epicytic folds; cytoplasm (c), micropore (encircled), cytoplasm of folds (*), groove (g), EF face of external cytomembrane (Ee), EF face of internal cytomembrane (Ei), IMP alignments (arrowhead), PF face of external cytomembrane (Pe), PF face of internal cytomembrane (Pi), plasma membrane (arrow), pores (white arrows). **B**. The base of the epicytic folds; cytoplasm of folds (*), deutomerite cytoplasm (c), duct (encircled), grooves (g), mucus (x), PF face of the external cytomembrane (Pe), pores (white arrows). **C**, **D**. The fracture of the epicytic fold; cytoplasm (c), EF of the external cytomembrane (Ee), EF face of the internal cytomembrane (Ei), PF face of the external cytomembrane (Pe), PF face of the internal cytomembrane (Pi), pores (white arrows), IMP alignments (arrowheads). **E**. The base of the fold with a duct opening outwards (arrow) to the groove; deutomerite cytoplasm (c), EF of the external cytomembrane (Ee), PF face of the external cytomembrane (Pe), PF face of the internal cytomembrane (Pi). **F**. The longitudinal fracture of the fold; cytoplasm (c), cytoplasm of fold (*), PF face of the external cytomembrane (Pe), PF face of the internal cytomembrane (Pi), pores (white arrows). **G**. The grooves (g) between the folds (ef); cytoplasm (c), mucus (x), numerous pores (some of them are shown by white arrows), PF face of the external cytomembrane (Pi). The inset shows a micropore and a pore of smaller size. **H**. A detail of the grove between folds (ef) showing the part of micropore (arrow) with vesicle (v); deutomerite cytoplasm (c). **I**. The top of the fold; EF face of the plasma membrane (Ep), IPM alignments (arrowheads), PF face of the external cytomembrane (Pe). The arrowhead in the circle shows the direction of shadowing.

**Figure 9:**
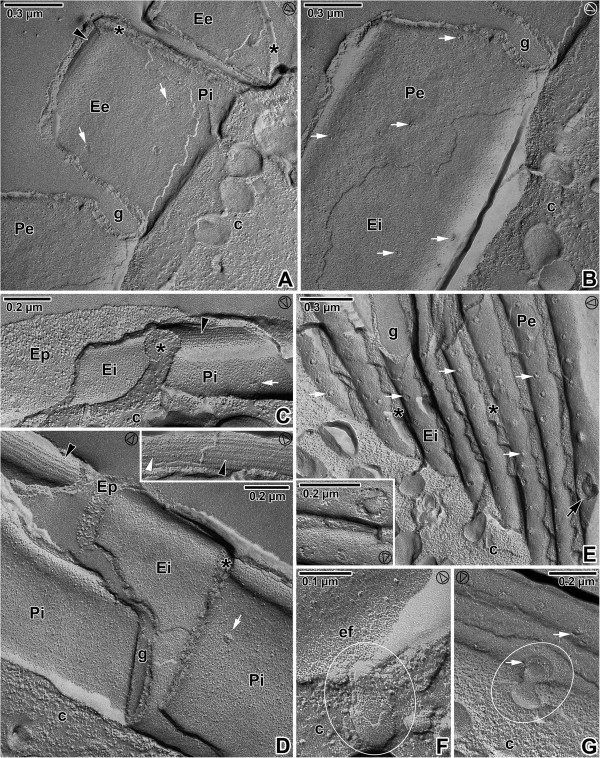
**Pellicle organisation in *Gregarina polymorpha *gamonts as revealed by the freeze-etching. A**. The general view of fractured epicytic folds; cytoplasm of folds (*), deutomerite cytoplasm (c), EF face of external cytomembrane (Ee), IMP alignments (arrowhead), groove (g), PF face of external cytomembrane (Pe), PF face of internal cytomembrane (Pi), pores (white arrows). **B**. The fractured epicytic fold; deutomerite cytoplasm (c), EF face of the internal cytomembrane (Ei), groove (g), PF face of the external cytomembrane (Pe), pores (white arrows). **C**. The fracture of the lower epicytic fold; cytoplasm of folds (*), deutomerite cytoplasm (c), EF face of the internal cytomembrane (Ei), EF face of the plasma membrane (Ep), IMP alignments (arrowhead), PF face of the internal cytomembrane (Pi), pore (white arrow). **D**. The fracture of higher epicytic folds; cytoplasm of folds (*), deutomerite cytoplasm (c), EF of the internal cytomembrane (Ei), EF of the plasma membrane (Ep), groove (g), IMP alignments (arrowhead), PF face of the internal cytomembrane (Pi), pore (white arrow). The inset shows the IMP alignments located on the EF face of the external cytomembrane (white arrowhead) and on the PF of the internal cytomembrane (arrowhead) at the top of the epicytic fold. **E**. The grooves (g) between epicytic folds with numerous pores (some of them shown by white arrows); cytoplasm of folds (*), deutomerite cytoplasm (c), EF face of the internal cytomembrane (Ei), PF face of the external cytomembrane (Pe). Note the large opened micropore (arrow). The inset shows a micropore and three pores of different sizes. **F**. The base of the epicytic fold (ef) with a duct (encircled); cytoplasm (c). **G**. The base of the epicytic folds showing the micropore connected to a vesicle (encircled); cytoplasm (c), pores (white arrows). The arrowhead in the circle shows the direction of shadowing.

**Figure 10:**
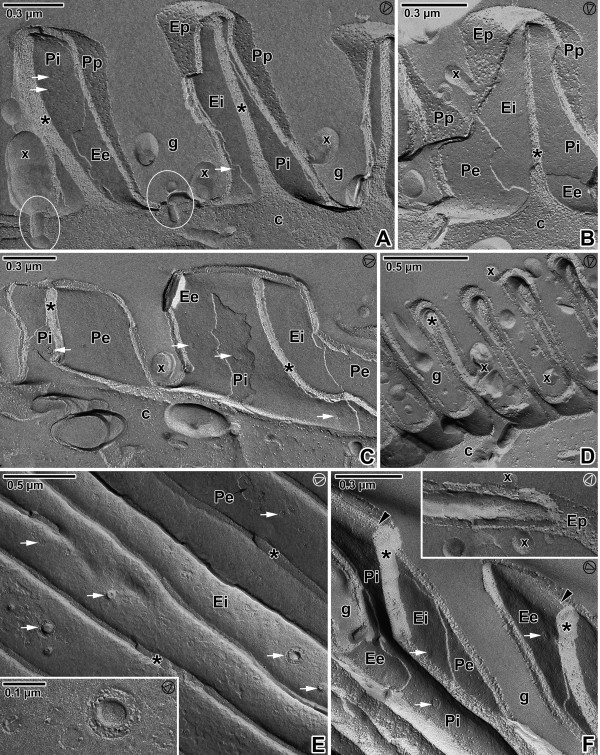
**Pellicle organisation in *Gregarina steini *gamonts as revealed by the freeze-etching. A–C**. Fractured epicytic folds; cytoplasm of epicytic folds (*), deutomerite cytoplasm (c), ducts (encircled), EF face of the external cytomembrane (Ee), EF face of the internal cytomembrane (Ei), EF face of the plasma membrane (Ep), groove (g), mucus (x), PF face of the external cytomembrane (Pe), PF face of the internal cytomembrane (Pi), PF face of the plasma membrane (Pp), pores (white arrows). **D**. General view of the epicytic folds showing the grooves (g) with mucus drops (x); cytoplasm of epicytic folds (*), deutomerite cytoplasm (c). **E**. The cytoplasmic face of the grooves between epicytic folds with micropores and numerous pores (some of them shown by white arrows); cytoplasm of epicytic folds (*), EF face of the internal cytomembrane (Ei), PF face of the external cytomembrane (Pe). The inset shows the detailed view of micropore and four pores of different sizes. **F**. Fractured epicytic folds showing their IMP alignments (arrowheads) located on the PF of the internal cytomembrane (Pi) and on the EF face of the external cytomembrane (Ee); cytoplasm of epicytic folds (*), EF face of internal cytomembrane (Ei), grooves (g), PF face of external cytomembrane (Pe), pores (white arrows). The inset shows EF of the plasma membrane (Ep) with mucus drops (x). The arrowhead in the circle shows the direction of shadowing.

**Figure 11:**
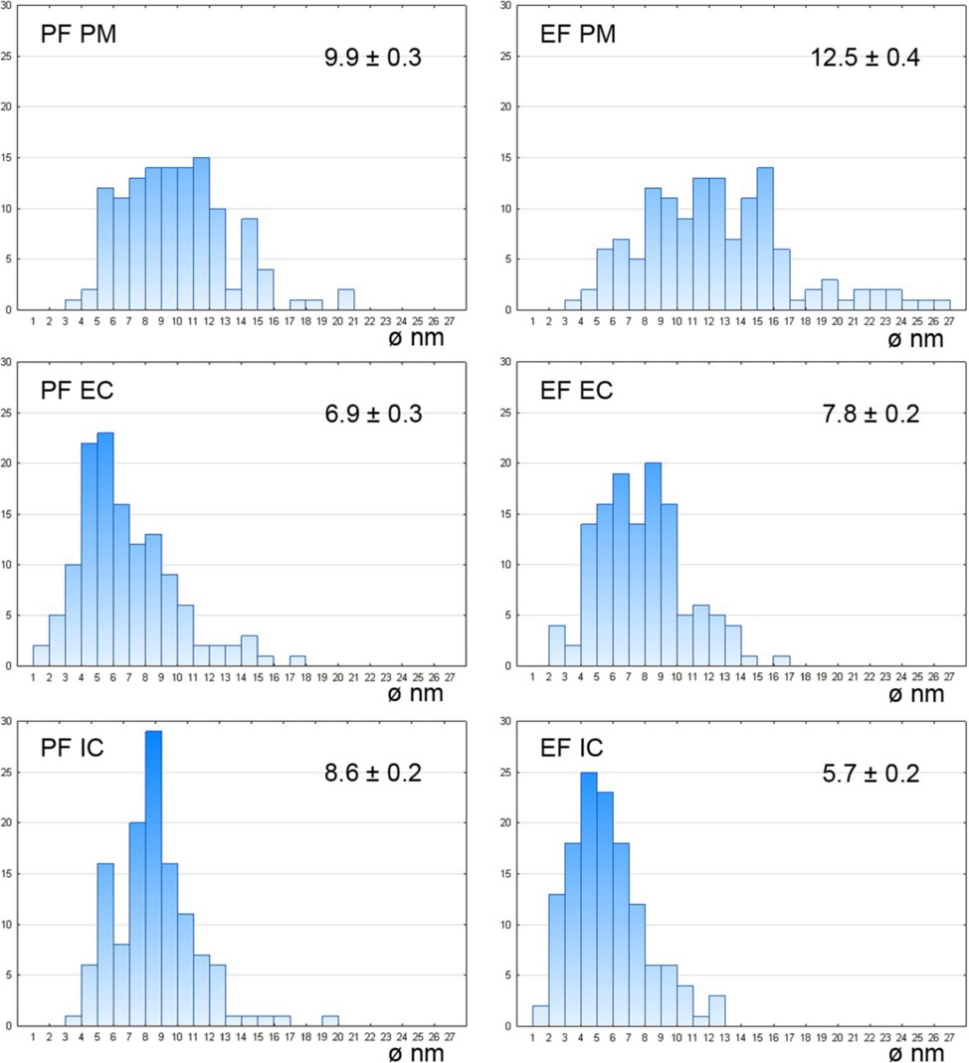
**Histograms illustrating the IMP size distribution in *Gregarina cuneata*; protoplasmic (PF) and exoplasmic fracture (EF) faces of the plasma membrane (PM), external cytomembrane (EC), internal cytomembrane (IC).** The mean diameter (nm) of IMP, with its standard error, is given for each. Ordinate - number of particles; abscissa - particle diameter in nm.

The folded pellicle was three-layered, composed of the superficial plasma membrane covering the entire gregarine and a middle lucent region, underlined by two distinct and tightly apposed membranes, i.e., external and internal cytomembrane, forming the inner membrane complex (IMC). Epicytic folds emerged from the peripheral ectoplasm bounding the endoplasm. Three types of associated structures were constantly present in each fold: an internal lamina, 12-nm filaments and rippled dense structures. The internal lamina, running under the IMC, not only linked the bases of epicytic folds but also bifurcated just beneath each fold, and its thinner part extended to the individual folds (Figures 5G, 6G and 7I). The localisation along with the organisation of internal lamina suggest its function in the stabilisation of individual folds as well their interconnection. The thickness of the internal lamina varied by species; i.e., 50–75 nm in *G. polymorpha* (Figure 6F–G), 17–30 nm in *G. cuneata* (Figure 5G–H) and 8–11 nm in *G. steini* (Figure 7H–I). The compact organisation of the internal lamina usually disappeared when reaching the region of 12-nm filaments. In fact, the 12-nm filaments seemed to be embedded in the widened area of the internal lamina (Figures 5G, 6G and 7I). The 12-nm filaments, exhibiting the properties of intermediate filaments, ran under the IMC along the longitudinal axis of each fold. Their numbers varied by species and developmental stage, i.e., in gamonts of *G. cuneata* up to 7 (Figure 5G) and in *G. polymorpha* up to 10 filaments were observed (Figure 6G), while in *G. steini* only 2–4 filaments could be seen (Figure 7I). Their number increased with fold maturation, i.e., new rising folds contained fewer filaments than the older and evidently higher epicytic folds. Rippled dense structures, located between the plasma membrane and IMC, appeared as electron-dense, triangle-shaped structures with base lying on the external cytomembrane and median running in between two adjacent 12-nm filaments. Their number varied by species and developmental stages, in correlation with the number of 12-nm filaments (Figures 5G, 6G and 7I). Unknown dense and usually half-moon-shaped structures underlined the 12-nm filaments at their cytoplasmic face in all gregarines. This structure achieved its maximum length in *G. polymorpha*, in which its ends were in obvious contact with the internal lamina extending to the top of the fold (Figure 6G). In *G. cuneata,* this structure was evidently shorter and thicker (Figure 5G), and in *G. steini* it was shortest and in some sections it was even reversed with its ends facing the apical top of the fold (Figure 7I). As the internal lamina lacked its typical compact look in this area, it is possible that the mentioned half-mooned structures represent its component. Careful analysis of the pellicle covering the lateral part of epicytic folds revealed novel thin filamentous connections interconnecting the IMC and the plasma membrane (Figures 5I, 6H and 7I).

In addition to structures restricted to the epicytic folds, an ectoplasmic network and rib-like myonemes, present to some degree in the deutomerite ectoplasm of all studied species, could be observed in some ultrathin sections. The ectoplasmic network contacting the bases of the epicytic folds was most prominent in fully matured gamonts of *G. cuneata* (Figure 5J–L), especially at the septum periphery or in areas with an obviously pleated pellicle (Figure 5K). This local pleating of the pellicle seemed to be the result of gregarine movement. The rib-like myonemes, running perpendicularly to the longitudinal axis of the gregarine and located beneath the deutomerite ectoplasm, were very distinct in gamonts of *G. cuneata* (Figure 5H and J) and *G. polymorpha* (Figure 6F), but were hard to detect and often absent in *G. steini*. The septum separating the protomerite from the deutomerite was well developed in all three species (Figures 5L, 6K and 7J). The micropores, interrupting the IMC, were sometimes seen in the grooves between the folds of *G. cuneata* (Figure 5J). In addition, ducts, appearing as elongated dense sacs passing through the pellicle and opening outwards, were often present in the ectoplasm of *G. cuneata* and *G. steini* gamonts (Figures 5J and 7L).

### Membranes as exposed by freeze etching

The freeze-etching technique confirmed the presence of three fracture planes in the pellicle of all analysed gregarines, corresponding to the three membranes, i.e., two cytomembranes (IMC) underlying the plasma membrane. The general aspects of the epicytic folds in freeze-fracture are shown in Figures 8, 9 and 10, and their architecture corresponded to the TEM observations. At the tip of each fold, double linear rows of tightly aligned intramembranous particles (IMP) were observed in both fractures of the external and internal cytomembranes. These rows seemed to correspond to the apical site of the 12-nm filaments and to the base of the triangle-shaped, rippled dense structures. The number of these lines roughly coincided with the number of 12-nm filaments. The freeze-etching approach proved to be a strong tool for visualisation of structures that are hardly documented on TEM micrographs including round micropores (110–130 nm in diameter) at the base of the grooves between the folds (Figures 8A, 8G, 9E, 9G and 10E) or smaller pores (35–50 nm in diameter) randomly distributed on the base or on the lateral side of the folds (Figures 8A–D, 8F-G, 9A–E, 9G, 10A, 10C and 10E–F), ducts and cisternae often connected these pores or to the pellicle (Figures 8B, 8E, 8H, 9F–G and 10A), as well as mucus drops often present in the grooves between the folds of *G. cuneata* (Figure 8B) and *G. steini* (Figure 10A–D and F).

**Figure 12:**
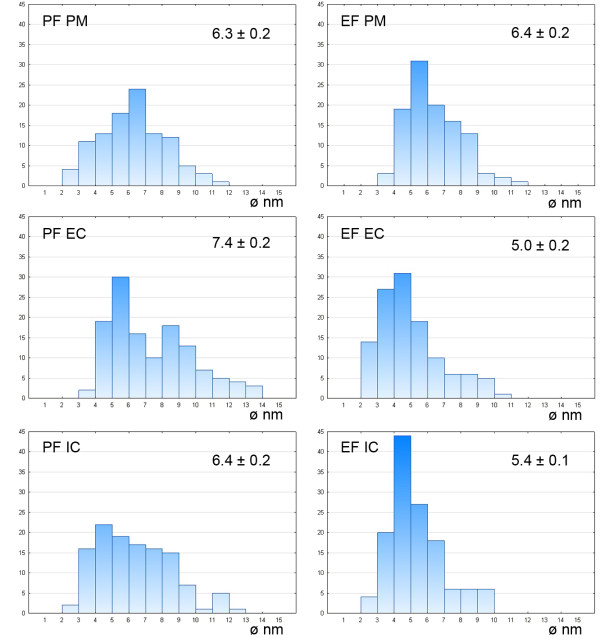
**Histograms illustrating the IMP size distribution in *Gregarina polymorpha*; protoplasmic (PF) and exoplasmic fracture (EF) faces of the plasma membrane (PM), external cytomembrane (EC), internal cytomembrane (IC).** The mean diameter (nm) of IMP, with its standard error, is given for each. Ordinate - number of particles; abscissa - particle diameter in nm.

**Figure 13:**
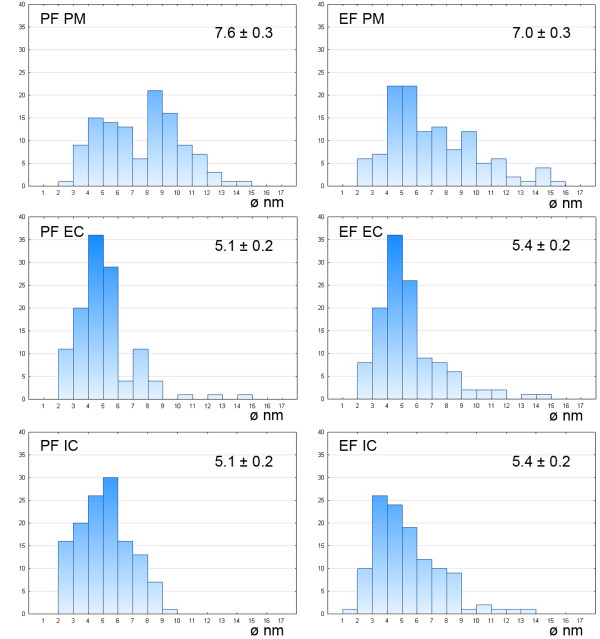
**Histograms illustrating the IMP size distribution in *Gregarina steini*; protoplasmic (PF) and exoplasmic fracture (EF) faces of the plasma membrane (PM), external cytomembrane (EC), internal cytomembrane (IC).** The mean diameter (nm) of IMP, with the standard error, is given for each. Ordinate - number of particles; abscissa - particle diameter in nm.

The densities of the IMP for the fracture faces of the plasma membrane and both cytomembranes are summarised in Table 5. The density of the IMP in membranes differed in analysed gregarines; in general, the values in *G. polymorpha* were significantly lower in comparison to *G. cuneata* and *G. steini*. In *G. cuneata* and *G. polymorpha*, the IMP density in the IMC was lower than in the plasma membrane. In *G. steini*, however, the IMP density in the plasma membrane was lower than those in the IMC. The IMP in all three membranes showed a high variability in their size distribution (see histograms in Figures 11, 12 and 13); nevertheless, only particles in a range of 6–14 nm were included in statistical calculations to obtain data comparable with those previously published on other apicomplexans. Membranes forming the pellicle in *G. cuneata*, especially the plasma membrane, were the most extraordinary given the wide range of the IMP size along with its distribution (Figure 11).

**Table 5:** A protein particle density in the fracture faces of the membranes of studied gregarines

**Species**		***Gregarina cuneata***		***Gregarina polymorpha***		***Gregarina steini***	
**Membrane**	**Face**	**Density = number of particles/μm**^**2 **^**(± SE)**	**Kp**	**Density = number of particles/μm**^**2 **^**(± SE)**	**Kp**	**Density = number of particles/μm**^**2 **^**(± SE)**	**Kp**
**Plasma membrane**	PF	2244 ± 283	0.81	1446 ± 158	0.59	2265 ± 154	1.27
	EF	2770 ± 96		2473 ± 147		1783 ± 233	
**External cytomembrane**	PF	1420 ± 190	1.13	602 ± 265	0.70	2588 ± 189	0.68
	EF	1260 ± 211		863 ± 202		3820 ± 211	
**Internal cytomembrane**	PF	1993 ± 253	1.33	1276 ± 200	1.57	2339 ± 132	1.24
	EF	1502 ± 273		814 ± 246		1886 ± 274	

The size of IMP is in range 6-14 nm.

*Kp* partition coefficient defined as the ratio of number of particles per μm^2^ in the PF face/number of particles per μm^2^ in the EF face.

*SE* standard error.

## Discussion

Gliding movement is a feature observed in a wide range of unicellular organisms including diatoms, flagellates, apicomplexan zoites, and gregarines. The speed of these organisms varies along with the special locomotive structures and is affected mostly by their physiological status and surrounding environmental conditions. The reported motility rates in Apicomplexa are usually in the range of 1–10 μm/s, and the maximal rate was observed in gregarines [34]. To minimise a potential effect of different environmental conditions on gregarine motility in this study, we took advantage of a naturally mixed infection with three *Gregarina* species occurring in the intestine of larval mealworms kept under laboratory conditions. In addition, the experimental part of this work, including the light microscopic observations on gliding and treatment of living gamonts with JAS and cytochalasin D, has been performed on suspensions consisting of all three species (often from a single host). Göhre [35] states that gregarines parasitising the intestine of larval *T. molitor* are distributed based on intestinal pH, i.e., *G. cuneata* inhabits a part of the intestine with pH 4.5–5.5, and *G. steini* (pH 5.5–8.2) inhabits a part of the intestine together with *G. polymorpha* (pH 6.4–7.5). Therefore, it could be expected that the mixed suspensions of gamonts are not preferable for motility assays, nevertheless, pH-restricted localisation of gregarines in mealworms applies only to attached trophozoites [36] not to gamonts that are usually found in luminal part of the mesenteron. During our observations, eugregarines isolated from one host were gliding at speeds ranging from 0.38 to 22.86 μm/s, while the highest speed was reached by gamonts of *G. polymorpha* and the lowest by *G. cuneata*. To explain these evident differences study focussed on structures that were generally considered to be responsible for gregarine gliding - epicytic folds and mucus. Longitudinal folds formed by the pellicle represent the most conspicuous feature differentiating eugregarine trophozoites and gamonts from the other apicomplexans. The presence of the swellings along the longitudinal epicytic folds of gliding gregarines suggests that these structures, pushed against the substrate, may provide the force for gliding [33], and the mucus seems to enhance the efficiency of their interaction with the substrate to produce gregarine forward movement. It is important, however, to mention that gregarine gamonts in performed experiments were able to move without any contact with the substrate when put in a drop of Ringer’s solution, i.e., for approximately 30 min, they were floating in a liquid until sinking to the surface of microscopic slide. These observations conflict with the supposed need of their contact with some solid matter [37]. Although some correlations can be found between speed and the number of epicytic folds, i.e., gamonts of *G. cuneata* are quipped by fewer folds (3.2–4.5) per square micrometer than small gamonts of *G. steini* (4.0–6.3) or similarly-sized gamonts of *G. polymorpha* (3.6–4.9), these differences were only slight and we do not consider them to significantly influence the gliding rate. More important, however, seem to be undulations of folds documented by SEM. In accordance with Vávra and Small [24], the folds of glutaraldehyde-fixed *G. cuneata* were almost linear, forming occasional rings of undulated folds, while the pellicle of *G. polymorpha* formed zones of undulated as well as almost linear folds, and the folds in *G. steini* were undulated in quite a regular pattern. Microscopic observations indicate that the lateral undulations of folds arise during gliding. Nevertheless, the question of whether epicytic folds might be responsible for gliding in gregarines can be satisfactorily answered only after careful analysis of all subcellular components forming the complicated pellicle in gregarines.

### The 'glideosome’ concept and eugregarine gliding

First, it must be highlighted that no similar structures resembling epicytic folds can be found on the surface of the zoite stage (e.g., sporozoites in gregarines or *Plasmodium,* tachyzoites of *Toxoplasma*), which are used to illustrate the mechanism of gliding motility in apicomplexans. The concept of the 'glideosome’ [4] describes apicomplexan zoites as actively entering host cells and moving by a substrate-dependent gliding motility, which requires coordinated interactions between parasite surface adhesins and its cytoskeleton. This machinery is considered an unusual form of eukaryotic locomotion. The so-called actomyosin motor, which is generally assumed to be embedded between the plasma membrane and the IMC, consists of immobilised unconventional myosins, short actin stubs, and TRAP-family invasins. This motor is expected to be oriented by subpellicular microtubules [38]. Micronemal proteins, inserted into the plasma membrane, are carried along the IMC by the motor and interact with the parasite substrate, or associate with a GPI-anchored protein interacting with the substrate, resulting in gliding [38].

Considering the possible application of this concept for eugregarine gliding, the first striking inconsistency is the fact that in the eugregarines analysed here, there are no subpellicular microtubules in the epicytic folds (confirmed also by negative results of immunolabelling). Thus, a question arises concerning the real motor in their motility. It could be expected that enigmatic 12-nm filaments, running under the IMC and exhibiting the properties of intermediate filaments [34,39], could support the actomyosin motor in a similar way. Longitudinal arrays of IMP found in the area of the 12-nm filaments and the rippled dense structures [11,22,30,40] are comparable to the lines of higher particle density overlaying the subpellicular microtubules in *Eimeria* or *Plasmodium* sporozoites [41,42]. Our data show, however, that the number of 12-nm filaments does not influence the speed of gregarine gliding (up to 7 filaments in *G. cuneata* vs. 10 filaments in *G. polymorpha* and maximally 4 filaments in *G. steini*), but rather seem to control the direction of movement. Indeed, despite folds equipped by a low number of 12-nm filaments, gamonts of *G. steini* glided with relatively high speed, but their gliding path was rather widely semi-circular than linear. The question remains whether apical rippled dense structures, with their base located at the external cytomembrane, serve as supporting elements interconnecting 12-nm filaments and the plasma membrane. Such speculation is supported by the existence of filamentous interconnections occasionally observed between their tips and the plasma membrane in ultrathin sections [39]. The half-moon-shaped dense structure underlining the 12-nm filaments could play the role of a 'skeleton’ reinforcing the tips of folds, which contact the substrate during gliding.

The apicomplexan gliding motility relies on the dynamic turnover of actin, the polymerisation of which is controlled by a number of regulators. A general model for the organisation of the apicomplexan actomyosin motor depicts actin filaments lying in the space between the parasite IMC and plasma membrane, parallel to the plasma membrane [43]. In gregarines, actin is generally expected to be localised in the epicytic folds [20,27]. Using a specific anti-actin antibody known to recognise the actin in *Toxoplasma* and *Plasmodium*, actin in all three gregarine species was localised. The unusual nature of apicomplexan actin, where its unpolymerised form seems to have an increased potential to form filaments relative to vertebrate actin, is discussed elsewhere [44]. In contrast to other eukaryotic cells, which maintain comparable amounts of globular and F-actin, in *Toxoplasma* more than 97% of actin is found in its globular form and in *Plasmodium*, where more F-actin may be recovered, the filamentous fraction appears to represent a collection of short polymers [27]. The apparent lack of visible, stable filaments in apicomplexans, however, does not fit eugregarines, in which the phalloidin labelling revealed that the presence of F-actin does not require filament-stabilising drugs [45,46]. Still, the results of JAS (a toxin that stabilizes actin filaments and induces actin polymerisation) and cytochalasin D (disrupts actin filaments and inhibits actin polymerisation) treatments are the clear evidence of an essential role of actin in eugregarine gliding. Both probes are membrane-permeable probes and thus suitable for examining actin dynamics in living cells, and are known to disrupt *Toxoplasma* motility and invasion [1]. The treatment of *Toxoplasma* tachyzoites with 1–2 μM JAS inhibited their gliding and cell invasion, but once the drug was removed the parasites were able to invade host cells [43]. Furthermore, JAS treatment increased the rate of *Toxoplasma* gliding [43], indicating that filaments are rate-limiting for motility and also caused frequent reversals of direction [3]. In *Eimeria* sporozoites, the inhibitor of actin polymerisation, cytochalasin B, reversibly inhibited the gliding; nevertheless, the bending was only slightly less [47]. In agreement with these studies, the treatment of living gregarines with JAS and cytochalasin D suspended their gliding motility, and they were able to recover after returning to normal physiological conditions in insect saline. In spite of high doses of both probes used in this study, prolonged incubations were necessary to inhibit gregarine gliding completely. Similar to *Toxoplasma* tachyzoites [43], JAS application led to an initial increased gliding activity, which gradually decreased until complete blocking. In contrast, reversals or changes of gliding direction and apical protrusion were not observed in gregarine gamonts. Interestingly, an enhanced deposition of actin, resembling an apical protrusion, occurred on the apical end of the migrating trophozoites of eugregarine *Ascogregarina*[48].

High concentrations of JAS can increase the density of actin filaments adjacent the plasma membrane [49]. Despite studies reporting competitive binding of JAS and phalloidin with F-actin [50], this work also revealed an increase in F-actin labelling in the cell cortex after a short treatment of living gamonts with JAS (corresponding to the period of their increased motility). These observations are supported by another study reporting that washing of cells before fixation and staining with phalloidin-TRITC to remove JAS revealed brighter staining of F-actin [51]. Prolonged treatment with JAS (until complete inhibition of gliding), however, resulted in an obvious decrease in F-actin labelling. These data concur with studies reporting that treatment of living cells with JAS causes a redistribution of their actin cytoskeleton, formation of F-actin aggregates and cell shape change, and can result in a patchy appearance of cortical actin [52]. Observations on gregarines during cytochalasin D treatment are supported by another study in which gliding was inhibited by cytochalasin B [53]. With regards to the Ca^++^ activation of the actomyosin system, it is interesting that the anti-psychotic drug trifluoperazine can inhibit gregarine gliding. This observation suggests that the calcium-binding protein calmodulin might affect motility even though extracellular Ca^++^ is not required [53].

Although the localisation of actin seems to be more diffuse in gregarines, myosin seems to be organised in longitudinal rows corresponding to epicytic folds similar to observations in *G. blaberae*[54]. The micrographs obtained with the anti-myosin antibody (smooth and skeletal, the whole antiserum from Sigma-Aldrich, Czech Republic) showed similar localization of myosin as obtained by Heintzelman with specific antibodies directed against the myosins A, B and F [20,27]. Nevertheless, the commercial antibody used in this study is developed for immunofluorescence and in the absence of conclusive results from Western Blotting this point needs further investigation. The presence of previously unreported tiny filamentous connections observed in some ultrathin sections between the plasma membrane and the IMC suggests that the actomyosin complex could be restricted to the lateral parts of the epicytic folds and, thus, this could be the source of the lateral undulations described above.

### Subcortical filamentous structures

In the course of environmental adaptations, the cortex of eugregarines became very rigid and, hence, they lost the wriggling ability of archigregarines. Nonetheless, when considering active movements of the protomerite, the cellular plasticity must be relatively high. Indeed, additional but very prominent forms of motility, such as bending, curving or shortening of the longitudinal axis and intense movements of the protomerite, commonly observed in eugregarines, were attributed to the presence of contractile elements entitled 'myocyte’ as early as one hundred years ago [37]. A network of intermediate filamentous proteins can be often found associated with the gregarine cortex. Early studies reporting the filamentous character of eugregarine 'myonemes’ suggest that they may consist of actin microfilaments [55], and recent studies confirmed them to be actin-rich [20]. An additional robust population of actin filaments, forming a series of annular (rib-like) myonemes girding the cell cortex, was reported from *G. polymorpha* but not seen in other apicomplexans [20,27]. Actin and myosin A were detected in both the epicytic folds and rib-like myonemes, while myosin B was exclusively restricted to folds [20]. Later on, a WD40 repeat-containing myosin designed myosin F, unique to the Apicomplexa and associated with rib-like myonemes, was reported in *G. polymorpha*[27]. Interestingly, the bending of the protomerite that is expected to be related to the ectoplasmic network and rib-like myonemes occurred in *G. polymorpha* and *G. cuneata* gamonts, which are indeed proven to possess these structures, while the stiff gamonts of *G. steini* missing the rib-like myonemes on ultrathin sections showed no shape changes during their rapid gliding. Despite the work reporting myosin- and actin-like proteins restricted to the vegetative stages of *G. blaberae*[54], the presence of these proteins was documented in both the gamonts as well as the trophozoites of *Gregarina* representatives [45,46]. Nevertheless, we do not exclude that there may exist some correlation between the abundance or form of actin and gregarine developmental stage.

### Shedding of mucous material

The next point that is worth noting with regard to the glideosome is the lack of micronemes in gregarine gamonts. Eugregarine gliding resembles the gliding motility in sporozoites of *Plasmodium*[56]. The trail left behind gliding ookinetes of *Plasmodium* was shown to correspond to the release of the Pbs25 and the circumsporozoite thrombospondin-related protein (CTRP) [57]. The material observed in the trail left after gliding eugregarines is generally designated as mucus, but more detailed biochemical analyses are needed to determine the exact composition. It could be expected the origin of this mucous material is related to numerous Golgi apparatuses present in the cytoplasm of gregarines [46,58,59]. The longest mucous trail was left behind gamonts of *G. polymorpha*. Similarly, Alcian blue staining at low pH, which proved to be very helpful to visualise mucous substances (i.e., glycosaminoglycans) [59,60], showed the highest amount of mucous material in the cytoplasm of *G. polymorpha* and lowest in *G. cuneata*. The results of this staining, however, conflict with the seemingly mucus-free surface of *G. polymorpha* and the abundant mucus-like drops covering the surface of *G. cuneata*, supported by the observations on the secretion of a mucus-like material in the grooves between *G. cuneata* folds [24]. Accepting the possibility that the increased production of mucus allows gregarines to glide with higher speeds, the differences in its viscosity could be the reason for mentioned observations on mucus secretion. Nevertheless, the origin of these drops remains unclear as no reliable conclusions about the chemical composition could be drawn from electron microscopic observations. Furthermore, the way of mucus secretion is not clear. It could be expected that openings or pore-like structures observed in the grooves between the folds might be related to mucus. Freeze-etching data further supported these speculations by the demonstration of numerous micropores in the pellicle lined by a collar and often in connections with some cisternae, vesicles or ducts, similar to those observed in *G. garnhami*[22]. Generally, the micropore is defined as an organelle formed by the apicomplexan pellicle, which is composed of two concentric rings (in transverse section), the inner of which corresponds with an invagination of the outer pellicle membrane. Micropores are assumed to have a feeding function but their real function is still poorly understood. A typical micropore was documented in ultrathin sections of the pellicle of *G. cuneata* (Figure 5J), and we assume that this structure corresponds to the pores revealed by the freeze-etching. Similar structures were observed in the pellicle of trophozoites of another *Gregarina* species [22,61] or in the pellicle of *Plasmodium* ookinete [62]. In addition, numerous tiny pore-like structures, located at the base and lateral side of epicytic folds, were found on the fractured faces of membranes, especially on the protoplasmic faces of IMC. The typical organisation of the proteins forming these structures proved them as pores.

### Intramembranous particles

We did not find any correlation between IMP density in membranes forming the pellicle and the gregarine gliding rate. Although the density of IMP, as well as the Kp, in studied gregarines differs, the overall values are highest in *G. steni* and lowest in *G. polymorpha*, both of which glide at high speed. Only IMP with their size ranging from 6 to 14 nm were used for statistical evaluation of their densities in order to get data comparable with those already published on other apicomplexans (Table 6). Most conspicuous differences in IMP densities can be seen between the values reported for IMC of *G. garnhami*[11] and our data, especially when considering that both studies focused on representatives of the same genus. It must be highlighted, however, that the statistical values differ considerably when including all visible IMP. A magnification of 56,000X used for statistics in this study allows visualisation of tiny IMP of 1 nm in diameter. That is why we included histograms illustrating the IMP size distribution in all analysed membranes to show their size variability among species as well as the frequency of particles with their sizes beyond this range. Considering the frequency of particles beyond the size range of 6–14 nm, especially in *G. cuneata*, it is questionable if this range set in previous studies really offers reliable data on IMP densities. An example is the differences in Kp for membranes when considering all detectable IMP in contrast to statistical values calculated for a size range 6–14 nm shown in Table 5; i.e., the Kp for the *G. cuneata* plasma membrane is 0.91 (in contrast to 0.81 for a set range of 6–14 nm), for the external cytomembrane is 0.93 (1.13) and for the internal cytomembrane is 1.49 (1.33); Kp for the *G. polymorpha* plasma membrane is 1.69 (0.59), for the external cytomembrane is 0.38 (0.70) and for the internal cytomembrane is 1.41 (1.57); and the Kp for the *G. steini* plasma membrane is 1.02 (1.27), for the external cytomembrane is 0.93 (0.68) and for the internal cytomembrane is 0.90 (1.24).

**Table 6:** **Densities of IMP (particles/μm**^**2**^**) in different apicomplexans**

**Species**	**Plasma membrane**		**External cytomembrane**		**Internal cytomembrane**	
	**EF**	**PF**	**PF**	**EF**	**EF**	**PF**
***Gregarina cuneata***	2770 ± 96	2244 ± 283	1420 ± 190	1260 ± 211	1502 ± 273	1993 ± 253
***Gregarina polymorpha***	2473 ± 147	1446 ± 158	602 ± 265	863 ± 202	814 ± 246	1276 ± 200
***Gregarina steini***	1783 ± 233	2265 ± 154	2588 ± 189	3820 ± 211	1886 ± 274	2339 ± 132
***Gregarina blaberae***^***1***^	977 ± 235	1469 ± 233	285 ± 39	133 ± 34	158 ± 72	297 ± 33
***Eimeria nieschulzi***^***2***^	218 ± 21	648 ± 73	2360 ± 133	29 ± 7	146 ± 31	1780 ± 97
***Plasmodium knowlesi***^***3***^	185 ± 25	2198 ± 528	17511 ± 228	38 ± 15	48 ± 28	574 ± 200

The size of IMP is in range 6-14 nm.

^1^Values taken from Schrével et al. [11].

^2^Values taken from Dubremetz and Topier [41].

^3^Values taken from McLaren et al. [63].

## Conclusions

Neither the general architectural features of the pellicle, including the number of epicytic folds or its subcellular components, nor the supramolecular organisation of the plasma membrane and IMC (density of IMP and their Kp) correlate with a gliding rate in eugregarines. Phalloidin and antibody labelling repeatedly confirmed the presence of actin and myosin restricted to the cell cortex. Moreover, the reaction of gregarines to the application of JAS and cytochalasin D serves as indirect proof of the importance of actin dynamic polymerisation during gregarine gliding. The location of the actomyosin complex seems to be restricted to the lateral parts of the epicytic folds rather than to their tips, as the number of 12-nm filaments and rippled dense structures running along their length does not influence the speed of gliding. The results of Alcian blue staining along with the mucous trail left behind gliding gamonts are the proof that the increased load of mucus in the cytoplasm correlates with gliding rate, as shown in *G. polymorpha* vs. *G. cuneata*, however, the viscosity of the mucus of the seemingly mucus-free surface of *G. polymorpha* needs further investigation. It is also worthy to highlight that despite the basic concept describing a substrate-dependent gliding in gregarines [31], for some period subsequent to drugs application to the cell suspension, gamonts were free-floating in a liquid lacking any contact with the substrate but with a significantly higher rate than exhibited during regular gliding.

Gregarines retained some ancestral features and are considered to be deep-branching apicomplexans. They evolved an enormous morphological and ecological diversity, and exhibit unique and novel adaptations to surrounding environment. Various gregarines parasitizing terrestrial and marine invertebrates not only exhibit diverse modes of locomotion (e.g., gliding in eugregarines with well-developed epicytic folds vs. bending, rolling or coiling known from marine archigregarines that probably evolved from hypertrophic zoite and retained subpellicular microtubules [9,17], and finally peristalsis-like movements observed in urosporidians), but even might use several mechanisms of cell motility depending on their actual physiological and environmental conditions. An understanding of the mechanism of gregarine motility and host cell invasion would offer significant insights into the parasitic strategy of apicomplexan parasites and evolution of obligate intracellular parasitism from free-living photosynthetic ancestors.

## Materials and methods

### Material collection

Larvae of the yellow mealworm, *Tenebrio molitor* Linnaeus, 1758 (Coleoptera, Tenebrionidae) infected with gregarines were obtained from colonies maintained in our laboratory. Gamonts of *Gregarina cuneata*, *G. polymorpha* and *G. steini* were collected from the intestinal lumen of naturally infected larvae. As all three eugregarine species can be simultaneously present in the larvae of *T. molitor*, experimental infections of larvae previously sterilised of gregarines were performed using infective oocysts in order to obtain a model infected with a single species for electron microscopic analyses. Protocols concerning experimental infection and following dissection of infected larvae are described elsewhere [8,45].

### Light microscopic observations on gliding motility

Single gamonts and gamonts associated in syzygy were removed from the host and placed on glass slides in Minimum Essential Medium [enriched with 3% bovine foetal serum with penicillin, streptomycin, amphotericin B and L-glutamine] (Sigma-Aldrich, Czech Republic). Incubation in this medium increased the viability of gregarines after isolation from host intestine. Light microscopic (LM) observations of gliding movement, orientation, and conformational changes were made. Results were confirmed by observations on gregarines incubated in Ringer’s insect physiological solution (pH 7.2) [64]. For speed calculations, short video records were taken using Bürker counting chamber. Individual cell speeds (in micrometers per second) were calculated from individual gregarine tracks by measurements of distances between initial and final positions covered over the time interval. The interval between selected recorded positions was normalised to 1 second using the ImageJ2x software developed at the National Institutes of Health.

For observations on mucus shedding, living gamonts were put on microscopic slides covered by a thin layer of microbiological agar, slightly moistened with Ringer’s solution and observed using phase contrast microscopy.

For treatment of gregarines with jasplakinolide (JAS; Invitrogen, Czech Republic) and cytochalasin D (Invitrogen, Czech Republic), living gamonts of *G. cuneata*, *G. steini* and *G. polymorpha* (a mixture of suspensions obtained from the guts of several hosts) were put on single cavity microscope glass slides with a drop of JAS or cytochalasin D in Ringer’s insect physiological solution (pH 7.2). The JAS was reconstituted in dimethyl sulfoxide (DMSO) to prepare a 1 mM stock solution and diluted in Ringer’s solution to prepare final working concentrations (5, 10, 20 and 30 μM JAS). Similarly, the 1 mM stock solution of cytochalasin D in DMSO was diluted in Ringer’s solution to obtain working concentrations (10, 20 and 30 μM cytochalasin D). Control assays of living gregarines were performed in pure Ringer’s solution as well as corresponding concentrations of DMSO in Ringer’s solution.

Cell suspensions, squash and/or wet smear preparations were investigated with the use of a motorised Olympus microscope BX61 equipped with Olympus DP71 digital camera and software (Olympus Stream Motion version 1.5.1).

### Mucus staining with alcian blue

Living gamonts were centrifuged at 10 000 × g for 30 minutes and subsequently fixed in freshly prepared 4% paraformaldehyde in phosphate buffered saline (PBS). After washing in 0.2 M PBS, cell suspensions were stained with Alcian blue (pH 1.3) for 1 hour, rinsed with 0.1 M hydrochloric acid and washed again in PBS [59]. For light microscopic analyses, the drops of stained cell suspension in PBS were dropped onto microscopic slides and covered by a cover slip.

### Electron microscopy

Cell suspensions were fixed overnight at 4°C in freshly prepared 2.5–3% glutaraldehyde in 0.2 M cacodylate buffer (pH 7.4). Procedures for sample processing for transmission (TEM) and scanning electron microscopy (SEM) follow Valigurová et al. [8,45]. Observations were made using a JEOL 1010 TEM and JEOL JSM-7401 F - Field emission scanning microscope.

### Freeze etching

Cell suspensions were fixed overnight at 4°C in freshly prepared 3% glutaraldehyde in 0.2 M cacodylate buffer (pH 7.4) followed by cryoprotection with 20% glycerol (w) treatment, concentrated on glass slides by using tweezers and put on the gold carrier. Specimens were then frozen in melting liquid nitrogen (-210°C), and for one cycle three carriers were mounted on a gold stand (subcooled in liquid nitrogen). Using the manipulator, the gold stand was transported into the working chamber (Freeze-etching system device, BAF 060 BAL-TEC) cooled to a temperature of -100°C at a pressure of 10^-5^ Pa. Subsequently, the specimens were cut and fractured with a microtome knife, etched (ice sublimation) for 2 minutes, and replicas were prepared by vacuum-deposition of platinum (the angle of evaporation was 45°, the thickness of layer 2.4 nm) and carbon (90° angle of evaporation, 22.2-nm thick layer) onto the frozen, fractured surface. The replicas were cleaned with 7% sodium hypochlorite and chromo-sulphuric acid to remove all the biological material, and washed in distilled water. The pieces of replica were mounted on copper grids and examined under a transmission electron microscope (Morgagni 268 D, FEI). Statistical evaluation of intramembranous particles (IMP) per a unit area (1 μm^2^) and histograms illustrating the IMP size distribution were done in ACC (Adaptive Contrast Control) developed by the Institute of Mathematics, Faculty of Mechanical Engineering, University of Technology, Brno. The nomenclature follows that proposed in Branton et al. [65] and used in Schrével et al. [11].

### Confocal laser scanning microscopy

Cell suspensions were washed in 0.2 M PBS, fixed for 15 minutes at room temperature in freshly prepared 4% paraformaldehyde in 0.2 M PBS, washed, and permeabilised for 15 minutes in 0.1-0.5% Triton X-100 (Sigma-Aldrich, Czech Republic). Protocols for direct staining of filamentous actin with phalloidin–tetramethylrhodamine B isothiocyanate (phalloidin-TRITC; Sigma-Aldrich, Czech Republic), as well as indirect immunofluorescent antibody (IFA) staining using the rabbit anti-myosin antibody (smooth and skeletal, the whole antiserum, Sigma-Aldrich, Czech Republic), the mouse monoclonal IgG anti-actin antibody that was raised against *Dictyostelium* actin (provided by Prof. Dominique Soldati-Favre) and mouse monoclonal anti-α-tubulin antibody (Clone B-5-1-2, Sigma-Aldrich, Czech Republic) follow Valigurová et al. [45,46]. Similarly, living gamonts treated for 10 and 150 minutes with 10 μM JAS were briefly washed in 0.2 M PBS and fixed for subsequent phalloidin labelling. Preparations were observed and documented using an Olympus IX80 microscope equipped with a laser-scanning FluoView 500 confocal unit (Olympus FluoView 4.3 software).

## Abbreviations

Act: Actin; CLSM: Confocal laser scanning microscopy; DMSO: Dimethyl sulfoxide; EF: Exoplasmic fracture; F-act: Filamentous actin; FITC: Fluorescein isothiocyanate; IFA: Indirect immunofluorescent assay; IMC: Inner membrane complex; IMP: Intramembranous particles; JAS: Jasplakinolide; Kp: Partition coefficient; LM: Light microscopy; Myo: Myosin; PF: Protoplasmic fracture; PBS: Phosphate buffered saline; SE: Standard error; SEM: Scanning electron microscopy; TEM: Transmission electron microscopy; TRITC: Tetramethylrhodamine B isothiocyanate.

## Competing interests

The authors declare that they have no competing interests.

## Authors’ contributions

AV conceived and designed the study, carried out the research, performed the experiments and microscopic analyses, and wrote the manuscript. JS contributed substantially to the conception and design of the study, interpretation of experimental data and to the writing of the manuscript. NV implemented freeze-etching techniques, performed related statistical analyses and data interpretation. NM assisted in the laboratory work, material collection and acquisition of data. All authors read and approved the final manuscript.

## References

[B1] Morrissette NS Sibley LD Cytoskeleton of apicomplexan parasites Microbiol Mol Biol Rev 2002 66 21 38 10.1128/MMBR.66.1.21-38.2002 11875126 PMC120781

[B2] Kappe SHI Buscaglia CA Bergman LW Coppens I Nussenzweig V Apicomplexan gliding motility and host cell invasion: overhauling the motor model Trends Parasitol 2004 20 13 16 10.1016/j.pt.2003.10.011 14700584

[B3] Wetzel DM Hakansson S Hu K Roos D Sibley LD Actin filament polymerization regulates gliding motility by apicomplexan parasites Mol Biol Cell 2003 14 396 406 10.1091/mbc.E02-08-0458 12589042 PMC149980

[B4] Opitz C Soldati D 'The glideosome’: a dynamic complex powering gliding motion and host cell invasion by *Toxoplasma gondii* Mol Microbiol 2002 45 597 604 10.1046/j.1365-2958.2002.03056.x 12139608

[B5] Kappe SHI Buscaglia CA Nussenzweig V *Plasmodium* sporozoite molecular cell biology Annu Rev Cell Dev Biol 2004 20 29 59 10.1146/annurev.cellbio.20.011603.150935 15473834

[B6] Santos JM Lebrun M Daher W Soldati D Dubremetz JF Apicomplexan cytoskeleton and motors: key regulators in morphogenesis, cell division, transport and motility Int J Parasitol 2009 39 153 162 10.1016/j.ijpara.2008.10.007 19028497

[B7] Barta JR Thompson RCA What is *Cryptosporidium*? Reappraising its biology and phylogenetic affinities Trends Parasitol 2006 22 463 468 10.1016/j.pt.2006.08.001 16904941

[B8] Valigurová A Hofmannová L Koudela B Vávra J An ultrastructural comparison of the attachment sites between *Gregarina steini* and *Cryptosporidium muris* J Eukaryotic Microbiol 2007 54 495 510 10.1111/j.1550-7408.2007.00291.x 18070327

[B9] Schrével J Goldstein S Kuriyama R Prensier G Vávra J Desportes I, Schrével J Biology of gregarines and their host-parasite interactions The gregarines: the early branching Apicomplexa 2013 Volume 1 Leiden, Netherlands: BRILL 26 196

[B10] Vivier E Devauchelle G Petitprez A Porchet-Hennere E Prensier G Schrével J Vinckier D Observations on comparative cytology in sporozoans. Part 1. The sperficial structures in vegetative forms Protistologica 1970 6 127 150

[B11] Schrével J Caigneaux E Gros D Philippe M The 3 cortical membranes of the gregarines. 1. Ultrastructural organization of *Gregarina blaberae* J Cell Sci 1983 61 151 174 6411745 10.1242/jcs.61.1.151

[B12] Schewiakoff W Über die Ürsache der fortschreitenden Bewegung der Gregarinen Z wiss Zool 1894 58 340 354

[B13] Mühl D Beitrag zur Kenntnis der Morphologie und Physiologie der Mehlwurm-Gregarinen Arch Protistenkd 1921 43 361 414

[B14] Richter IE Bewegungsphysiologische Untersuchungen an polycystiden Gregarinen unter Anwendung des Mikrozeitrafferfilmes Protoplasma 1959 51 197 241 10.1007/BF01252489

[B15] King C Sleep J Modelling the mechanism of gregarine gliding using bead translocation J Eukaryot Microbiol 2005 52 7S 27S

[B16] Vivier E Schrével J Etude au microscope electronique dune gregarine du genre *Selenidium* parasite de *Sabellaria alveolata* L J De Microscopie 1964 3 651 670

[B17] Schrével J Biological and ultrastructural observations on Selenidiidae and their relation to systematics of gregarines J Protozool 1971 18 448 470 10.1111/j.1550-7408.1971.tb03355.x

[B18] Baines I King CA Demonstration of actin in the protozoan *Gregarina* Cell Biol Int Reports 1989 13 679 686 10.1016/0309-1651(89)90044-1 2509082

[B19] Ghazali M Philippe M Deguercy A Gounon P Gallo JM Schrével J Actin and spectrin-like (Mr = 260-240 000) proteins in gregarines Biol Cell 1989 67 173 184

[B20] Heintzelman MB Actin and myosin in *Gregarina polymorpha* Cell Motil Cytoskeleton 2004 58 83 95 10.1002/cm.10178 15083530

[B21] Reger JF The fine structure of the gregarine *Pyxinoides balani* parasitic in the barnacle *Balanus tintinnabulum* J Protozool 1967 14 488 497 10.1111/j.1550-7408.1967.tb02034.x 4963675

[B22] Walker MH Lane NJ Lee WM Freeze-fracture studies on the pellicle of the eugregarine, *Gregarina garnhami* (Eugregarinida, Protozoa) J Ultrastruct Res 1984 88 66 76 10.1016/S0022-5320(84)90182-5

[B23] Korn H Ruhl H Comparative study of ultrastructure of *Gregarina polymorpha* and *Gregarina cuneata* (Sporozoa, Gregarinida) Z Parasitenkd - Parasitol Res 1972 39 285 301 10.1007/BF00329092 4628166

[B24] Vávra J Small EB Scanning electron microscopy of gregarines (Protozoa, Sporozoa) and its contribution to the theory of gregarine movement J Protozool 1969 16 745 757 10.1111/j.1550-7408.1969.tb02338.x 4983023

[B25] Valigurová A Koudela B Morphological analysis of the cellular, interactions between the eugregarine *Gregarina garnhami* (Apicomplexa) and the epithelium of its host, the desert locust *Schistocerca gregaria* Eur J Protistol 2008 44 197 207 10.1016/j.ejop.2007.11.006 18304787

[B26] Vivier E L’ organisation ultrastructurale corticale de la grégarine *Lecudina pellucida*; ses rapports avec l’ alimentation et la locomotion J Protozool 1968 15 230 246 10.1111/j.1550-7408.1968.tb02115.x

[B27] Heintzelman MB Mateer MJ GpMyoF, a WD40 repeat-containing myosin associated with the myonemes of *Gregarina polymorpha* J Parasitol 2008 94 158 168 10.1645/GE-1339.1 18372636

[B28] Heintzelman MB Schwartzman JD Characterization of myosin-A and myosin-C: two class XIV unconventional myosins from *Toxoplasma gondii* Cell Motil Cytoskeleton 1999 44 58 67 10.1002/(SICI)1097-0169(199909)44:1<58::AID-CM5>3.0.CO;2-R 10470019

[B29] Foth BJ Goedecke MC Soldati D New insights into myosin evolution and classification Proc Nat Acad Sci U S A 2006 103 3681 3686 10.1073/pnas.0506307103 PMC1533776 16505385

[B30] Dallai R Talluri MV Freeze-fracture study of the gregarine trophozoite: I the top of the epicyte folds Boll Zool 1983 50 235 244 10.1080/11250008309439448

[B31] King CA Cell-surface interaction of the protozoan gregarina with concanavalin-a beads - implications for models of gregarine gliding Cell Biol Int Rep 1981 5 297 305 10.1016/0309-1651(81)90228-9 6783325

[B32] Walker MH Mackenzie C Bainbridge SP Orme C Study of the structure and gliding movement of *Gregarina garnhami* J Protozool 1979 26 566 574 10.1111/j.1550-7408.1979.tb04197.x

[B33] Mackenzie C Walker MH Substrate contact, mucus, and eugregarine gliding J Protozool 1983 30 3 8 10.1111/j.1550-7408.1983.tb01024.x

[B34] King CA Cell motility of sporozoan protozoa Parasitol Today 1988 4 315 319 10.1016/0169-4758(88)90113-5 15463014

[B35] Göhre E Untersuchungen über den plasmatischen Feinbau der Gregarinen, mit besonderer Berücksichtigung der Sexualitätsverhältnisse Arch Protistenkd 1943 96 295 324

[B36] Weiser J Nemoci hmyzu 1966 Praha: Nakladatelství Československé akademie věd

[B37] Crawley H The Movements of gregarines Proc Acad Nat Sci Philadelphia 1905 57 89 99

[B38] Dubremetz JF Garcia-Reguet N Conseil V Fourmaux MN Apical organelles and host-cell invasion by Apicomplexa Int J Parasitol 1998 28 1007 1013 10.1016/S0020-7519(98)00076-9 9724870

[B39] Schrével J Philippe M Kreier JP The gregarines Parasitic Protozoa second edition 1993 Vol. 4 San Diego, United States: Academic Press, Inc. edition 133 245

[B40] Dallai R Talluri MV Evidence for septate junctions in the syzygy of the protozoan *Gregarina plymorpha* (Protozoa, Apicomplexa) J Cell Sci 1988 89 217 224

[B41] Dubremetz JF Torpier G Freeze fracture study of the pellicle of an eimerian sporozoite (Protozoa, Coccidia) J Ultrastruct Res 1978 62 94 109 10.1016/S0022-5320(78)90012-6 418187

[B42] Dubremetz JF Torpier G Maurois P Prensier G Sinden R Structure de la pellicule du sporozoite de *Plasmodium yoelii*: étude par cryofracture C R Acad Sci Paris 1979 Ser. D 623 626

[B43] Shaw MK Tilney LG Induction of an acrosomal process in *Toxoplasma gondii*: visualization of actin filaments in a protozoan parasite Proc Nat Acad Sci U S A 1999 96 9095 9099 10.1073/pnas.96.16.9095 PMC17738 10430901

[B44] Olshina MA Wong W Baum J Holding back the microfilamentu - structural insights into actin and the actin-monomer-binding proteins of apicomplexan parasites Iubmb Life 2012 64 370 377 10.1002/iub.1014 22454107

[B45] Valigurová A Michalková V Koudela B Eugregarine trophozoite detachment from the host epithelium via epimerite retraction: fiction or fact? Int J Parasitol 2009 39 1235 1242 10.1016/j.ijpara.2009.04.009 19460380

[B46] Valigurová A Sophisticated adaptations of *Gregarina cuneata* (Apicomplexa) feeding stages for epicellular parasitism PLoS ONE 2012 7 e42606 10.1371/journal.pone.0042606 22900033 PMC3416826

[B47] Russell DG Sinden RE The role of the cytoskeleton in the motility of coccidian sporozoites J Cell Sci 1981 50 345 359 7033252 10.1242/jcs.50.1.345

[B48] Chen WJ Fan-Chiang MH Directed migration of *Ascogregarina taiwanensis* (Apicomplexa: Lecudinidae) in its natural host *Aedes albopictus* (Diptera: Culicidae) J Eukaryot Microbiol 2001 48 537 541 10.1111/j.1550-7408.2001.tb00189.x 11596918

[B49] Bubb MR Spector I Beyer BB Fosen KM Effects of jasplakinolide on the kinetics of actin polymerization. An explanation for certain *in vivo* observations J Biol Chem 2000 275 5163 5170 10.1074/jbc.275.7.5163 10671562

[B50] Bubb MR Senderowicz AM Sausville EA Duncan KL Korn ED Jasplakinolide, a cytotoxic natural product, induces actin polymerization and competitively inhibits the binding of phalloidin to F-actin J Biol Chem 1994 269 14869 14871 8195116

[B51] Ayscough KR Endocytosis and the development of cell polarity in yeast require a dynamic F-actin cytoskeleton Curr Biol 2000 10 1587 1590 10.1016/S0960-9822(00)00859-9 11137010

[B52] Posey SC Bierer BE Actin stabilization by jasplakinolide enhances apoptosis induced by cytokine deprivation J Biol Chem 1999 274 4259 4265 10.1074/jbc.274.7.4259 9933626

[B53] King CA Lee K Effect of trifluoperazine and calcium ions on gregarine gliding Experientia 1982 38 1051 1052 10.1007/BF01955361

[B54] Ghazali M Schrével J Myosin-like protein (M(r)175,000) in *Gregarina blaberae* J Eukaryotic Microbiol 1993 40 345 354 10.1111/j.1550-7408.1993.tb04927.x 8508173

[B55] Hildebrand HF Electron-microscopic investigation on evolution stages of trophozoite of *Didymophyes gigantea* (Sporozoa, Gregarinida). 3. The fine structure of the epicyte with emphasis on the contractile elements Z Parasitenkd 1980 64 29 46 10.1007/BF00927055 6784366

[B56] Vanderberg JP Studies on the motility of *Plasmodium* sporozoites J Protozool 1974 21 527 537 10.1111/j.1550-7408.1974.tb03693.x 4138523

[B57] Lecona AN Rodriguez MH Argotte RS Alvarado A Rodriguez MC *Plasmodium berghei* ookinetes glide and release Pbs25 and circumsporozoite thrombospondin-related protein on solid surface substrata J Parasitol 2010 96 216 218 10.1645/GE-2193.1 19747017

[B58] Desportes I Ultrastructure et développement des grégarines du genre *Stylocephalus* Ann Sci Nat Zool Paris 1969 12 31 96

[B59] Schrével J Polysaccharides of cell-surface of gregarines (Protozoa Parasites). 1. Ultrastructure and cytochemistry J Microscopy-Oxford 1972 15 21 40

[B60] Schrével J Gros D Monsigny M Cytochemistry of cell glycoconjugates Prog Histochem Cytochem 1981 14 1 269 10.1267/ahc.14.1 6175992

[B61] Talluri MV Dallai R Freeze-fracture study of the gregarine trophozoite: II. Evidence of "rosette" organization on cytomembranes in relation with micropore structure Boll Zool 1983 50 247 256 10.1080/11250008309439449

[B62] Raibaud A Lupetti P Paul RE Mercati D Brey PT Sinden RE Heuser JE Dallai R Cryofracture electron microscopy of the ookinete pellicle of *Plasmodium gallinaceum* reveals the existence of novel pores in the alveolar membranes J Struct Biol 2001 135 47 57 10.1006/jsbi.2001.4396 11562165

[B63] McLaren DJ Bannister LH Trigg PI Butcher GA Freeze fracture studies on the interaction between the malaria parasite and the host erythrocyte in *Plasmodium knowlesi* infections Parasitology 1979 79 125 139 10.1017/S0031182000052021 120521

[B64] Belton P Grundfest H Potassium activation and K spikes in muscle fibers of the mealworm larva (*Tenebrio molitor*) Am J Physiol 1962 203 588 594 13866990 10.1152/ajplegacy.1962.203.3.588

[B65] Branton D Bullivant S Gilula NB Karnovsky MJ Moor H Muhlethaler K Northcote DH Packer K Satir B Satir P Freeze etching nomenclature Science 1975 190 54 56 10.1126/science.1166299 1166299

